# Skd3 (human ClpB) is a potent mitochondrial protein disaggregase that is inactivated by 3-methylglutaconic aciduria-linked mutations

**DOI:** 10.7554/eLife.55279

**Published:** 2020-06-23

**Authors:** Ryan R Cupo, James Shorter

**Affiliations:** 1 Department of Biochemistry and Biophysics, Perelman School of Medicine at the University of PennsylvaniaPhiladelphiaUnited States; 2 Pharmacology Graduate Group, Perelman School of Medicine at the University of PennsylvaniaPhiladelphiaUnited States; Texas Children's HospitalUnited States; Johns Hopkins University School of MedicineUnited States

**Keywords:** disaggregase, AAA+ protein, protein misfolding, Skd3, Hsp78, Hsp104, Human

## Abstract

Cells have evolved specialized protein disaggregases to reverse toxic protein aggregation and restore protein functionality. In nonmetazoan eukaryotes, the AAA+ disaggregase Hsp78 resolubilizes and reactivates proteins in mitochondria. Curiously, metazoa lack Hsp78. Hence, whether metazoan mitochondria reactivate aggregated proteins is unknown. Here, we establish that a mitochondrial AAA+ protein, Skd3 (human ClpB), couples ATP hydrolysis to protein disaggregation and reactivation. The Skd3 ankyrin-repeat domain combines with conserved AAA+ elements to enable stand-alone disaggregase activity. A mitochondrial inner-membrane protease, PARL, removes an autoinhibitory peptide from Skd3 to greatly enhance disaggregase activity. Indeed, PARL-activated Skd3 solubilizes α-synuclein fibrils connected to Parkinson’s disease. Human cells lacking Skd3 exhibit reduced solubility of various mitochondrial proteins, including anti-apoptotic Hax1. Importantly, Skd3 variants linked to 3-methylglutaconic aciduria, a severe mitochondrial disorder, display diminished disaggregase activity (but not always reduced ATPase activity), which predicts disease severity. Thus, Skd3 is a potent protein disaggregase critical for human health.

## Introduction

Protein aggregation and aberrant phase transitions are elicited by a variety of cellular stressors and can be highly toxic (Chuang et al., 2018; Eisele et al., 2015; Guo et al., 2019). To counter this challenge, cells have evolved specialized protein disaggregases to reverse aggregation and restore resolubilized proteins to native structure and function (Shorter, 2017; Shorter and Southworth, 2019). Indeed, protein disaggregases are conserved across all domains of life, with orthologues of Hsp104, a ring-shaped hexameric AAA+ protein, powering protein disaggregation and reactivation (as opposed to degradation) in eubacteria and nonmetazoan eukaryotes (Glover and Lindquist, 1998; Goloubinoff et al., 1999; Queitsch et al., 2000; Shorter, 2008). In nonmetazoan eukaryotes, Hsp104 functions in the cytoplasm and nucleus (Parsell et al., 1994; Tkach and Glover, 2008; Wallace et al., 2015), whereas the closely-related AAA+ disaggregase, Hsp78, resolubilizes and reactivates proteins in mitochondria (Krzewska et al., 2001; Schmitt et al., 1996). Curiously, at the evolutionary transition from protozoa to metazoa both Hsp104 and Hsp78 are lost and are subsequently absent from all animal species (Erives and Fassler, 2015). This loss of Hsp104 and Hsp78 is perplexing given that toxic protein aggregation remains a major challenge in metazoa (Eisele et al., 2015). Indeed, it is even more baffling since ectopic expression of Hsp104 is well tolerated by animal cells and can be neuroprotective in animal models of neurodegenerative disease (Cushman-Nick et al., 2013; Dandoy-Dron et al., 2006; Jackrel et al., 2014; Lo Bianco et al., 2008; Perrin et al., 2007; Satyal et al., 2000; Vacher et al., 2005; Yasuda et al., 2017).

Metazoa may partially compensate for the absence of Hsp104 activity in the cytoplasm and nucleus with alternative general protein-disaggregase systems, such as Hsp110, Hsp70, Hsp40, and small heat-shock proteins (Duennwald et al., 2012; Mattoo et al., 2013; Nillegoda et al., 2015; Scior et al., 2018; Shorter, 2011; Shorter, 2017; Torrente and Shorter, 2013) as well as client-specific disaggregases in the cytoplasm such as nuclear-import receptors (Guo et al., 2019; Guo et al., 2018; Niaki et al., 2020; Yoshizawa et al., 2018). However, Hsp110 is not found in mitochondria (Voos and Röttgers, 2002). Thus, it continues to remain uncertain whether, in the absence of Hsp78, metazoan mitochondria harbor a disaggregase that solubilizes and reactivates aggregated proteins.

**Figure 1.**
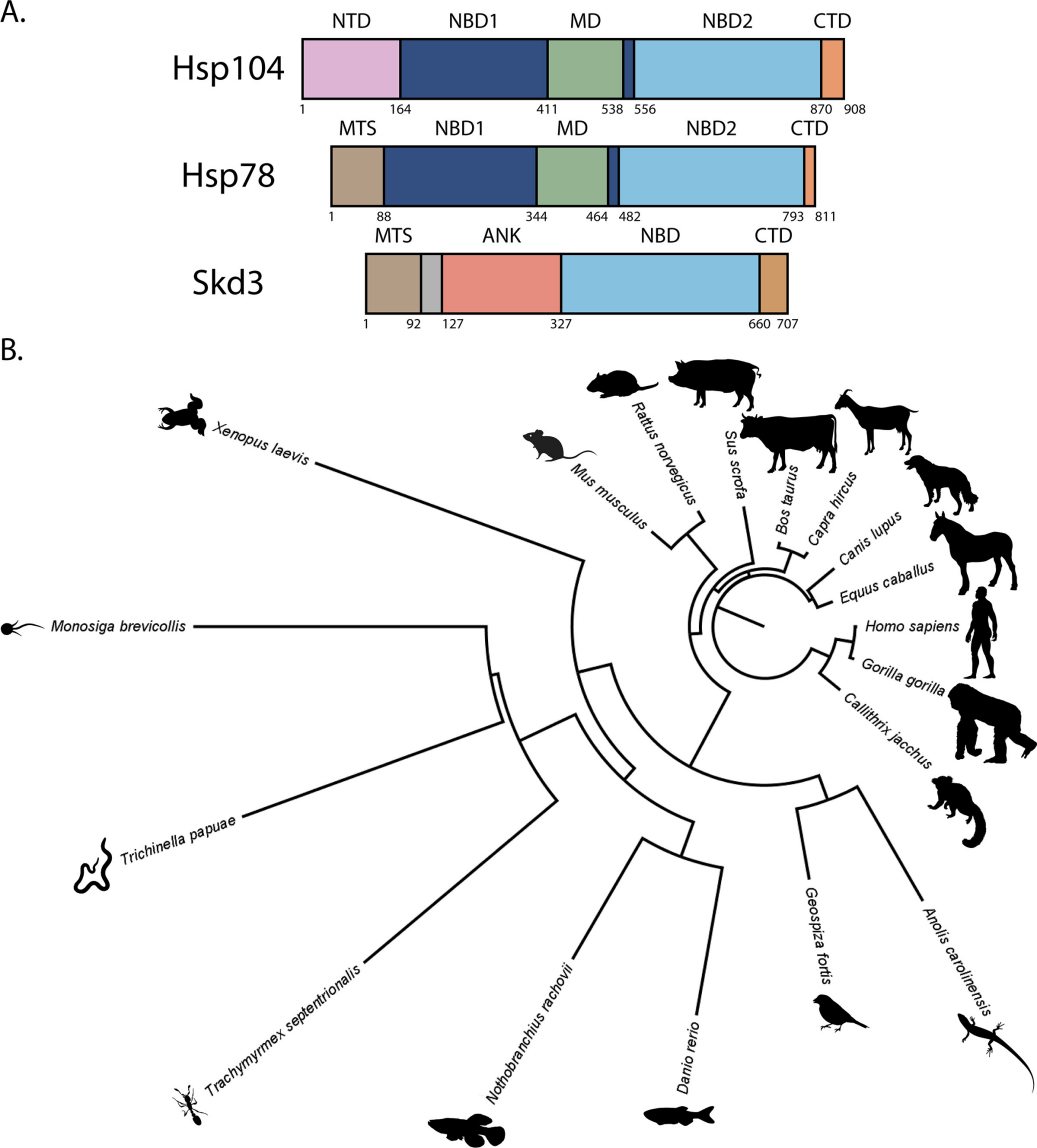
Skd3 is homologous to Hsp104 and Hsp78 and is conserved across diverse metazoan lineages. (**A**) Domain map depicting *S. cerevisiae* Hsp104, *S. cerevisiae* Hsp78, and *H. sapiens* Skd3. Hsp104 is composed of a N-terminal domain (NTD), nucleotide-binding domain 1 (NBD1), middle domain (MD), nucleotide-binding domain 2 (NBD2), and C-terminal domain (CTD). Hsp78 is composed of a mitochondrial-targeting signal (MTS), NBD1, MD, NBD2, and CTD. Skd3 is composed of a MTS, a short hydrophobic stretch of unknown function, an ankyrin-repeat domain (ANK) containing four ankyrin repeats, an NBD that is homologous to Hsp104 and Hsp78 NBD2, and a CTD. (**B**) Phylogenetic tree depicting a Clustal Omega alignment of Skd3 sequences from divergent metazoan lineages and the protozoan *Monosiga brevicollis*. The alignment shows conservation of Skd3 across diverse species and shows high similarity between mammalian Skd3 proteins.

Here, we investigate whether Skd3 (encoded by human *CLPB*) might act as a mitochondrial protein disaggregase in metazoa (Figure 1a). Skd3 is a ubiquitously expressed, mitochondrial AAA+ protein of poorly-defined function, which is related to Hsp104 and Hsp78 via its HCLR clade AAA+ domain (Figure 1a and Figure 1—figure supplement 1; Erzberger and Berger, 2006; Périer et al., 1995; Seraphim and Houry, 2020). Skd3 appears to play an important role in maintaining mitochondrial structure and function (Chen et al., 2019). Curiously, Skd3 first appears in evolution alongside Hsp104 and Hsp78 in choanoflagellates, a group of free-living unicellular and colonial flagellate eukaryotes that are the closest extant protozoan relatives of animals (Figure 1b and Supplementary file 1; Brunet and King, 2017; Erives and Fassler, 2015). During the complex evolutionary transition from protozoa to metazoa, Skd3 is retained, whereas Hsp104 and Hsp78 are lost (Erives and Fassler, 2015). Indeed, Skd3 is conserved in many metazoan lineages (Figure 1a,b, Figure 1—figure supplement 1, and Supplementary file 1; Erives and Fassler, 2015).

**Figure 1—figure supplement 1.**
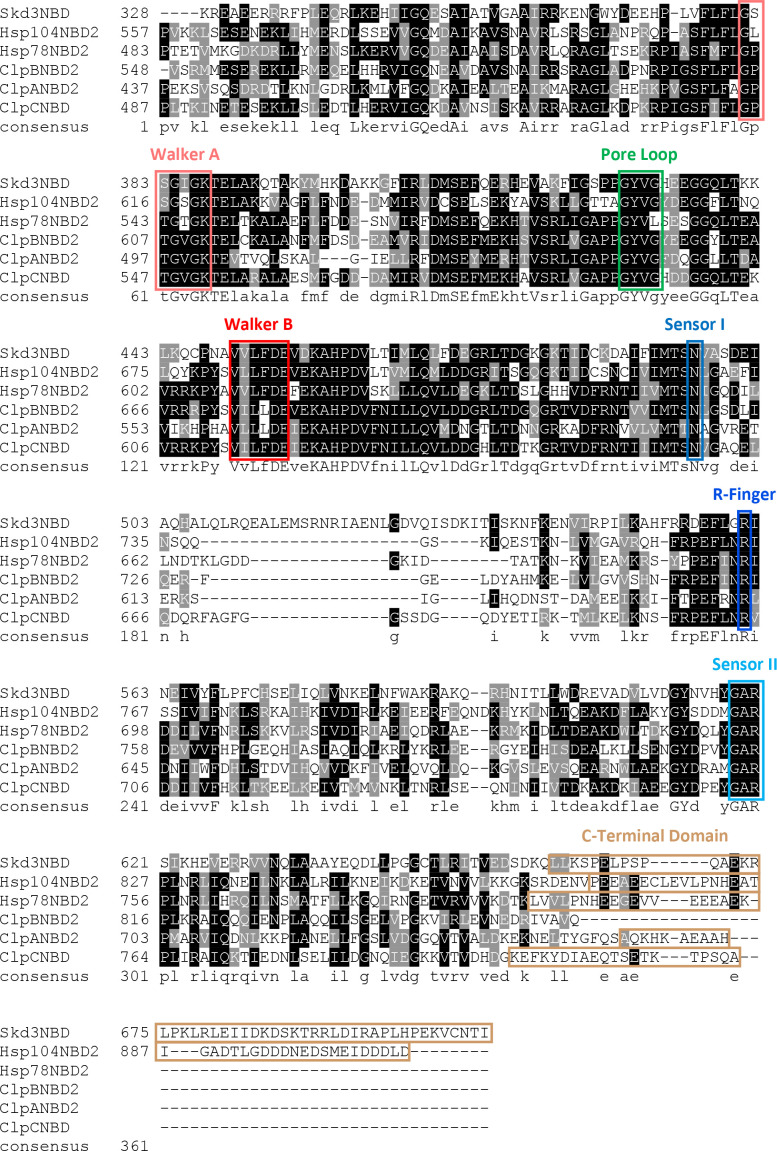
Skd3 NBD alignment to other AAA+ proteins reveals high similarity to Hsp104 and Hsp78. Alignment of the NBD from *H. sapiens* Skd3 and NBD2 from *S. cerevisiae* Hsp104, *S. cerevisiae* Hsp78, *E. coli* ClpB, *E. coli* ClpA, and *S. aureus* ClpC. Alignments were constructed using Clustal Omega. Bottom row shows consensus sequence of alignment. Highlighted in red are the Walker A and Walker B motifs. Highlighted in green are the Pore-Loop motifs. Highlighted in blue are the Sensor I, Sensor II, and Arginine-Finger motifs.

Skd3 is comprised of a mitochondrial-targeting signal (MTS), followed by a short hydrophobic stretch, an ankyrin-repeat domain (ANK), an AAA+ nucleotide-binding domain (NBD), and a small C-terminal domain (CTD) (Figure 1a). The Skd3 NBD closely resembles NBD2 of Hsp104 and Hsp78 (Figure 1a and Figure 1—figure supplement 1). Aside from this similarity, Skd3 is highly divergent from Hsp104 and Hsp78 (Figure 1a and Figure 1—figure supplement 1; Torrente and Shorter, 2013). For example, Skd3, Hsp104, and Hsp78 all have short CTDs, but these are divergent with the Skd3 CTD being basic compared to the more acidic Hsp104 and Hsp78 CTDs (Figure 1—figure supplement 1). Moreover, the other domains in Hsp104 (N-terminal domain [NTD], NBD1, and middle domain [MD]) and Hsp78 (NBD1 and MD) are not found in Skd3 (Figure 1a and Figure 1—figure supplement 1). In their place, is an ankyrin-repeat domain (Figure 1a), which interestingly is an important substrate-binding domain of another protein disaggregase, chloroplast signal recognition particle 43 (cpSRP43) (Jaru-Ampornpan et al., 2013; Jaru-Ampornpan et al., 2010; McAvoy et al., 2018; Nguyen et al., 2013).

Importantly, mutations in the Skd3 ankyrin-repeat domain and NBD are linked to the rare, but severe mitochondrial disorder, 3-methylglutaconic aciduria, type VII (MGCA7) (Capo-Chichi et al., 2015; Kanabus et al., 2015; Kiykim et al., 2016; Pronicka et al., 2017; Saunders et al., 2015; Wortmann et al., 2016; Wortmann et al., 2015). MGCA7 is an autosomal recessive metabolic disorder that presents with increased levels of 3-methylglutaconic acid (3-MGA), neurologic deterioration, and neutropenia (Wortmann et al., 2016). Typically, patients present with infantile onset of a progressive encephalopathy with movement abnormalities and delayed psychomotor development (Wortmann et al., 2016). Other common, but variable, phenotypes include cataracts, seizures, and recurrent infections (Wortmann et al., 2016). These issues can be severe with afflicted infants typically only living for a few weeks or months (Wortmann et al., 2016). Patients may also present with more moderate phenotypes, including neutropenia, hypotonia, spasticity, movement abnormalities, epilepsy, and intellectual disability (Wortmann et al., 2016). Mildly affected individuals have no neurological problems, normal life expectancy, but present with neutropenia (Wortmann et al., 2016). There is no cure and no effective therapeutics for severe or moderate forms of MGCA7. Moreover, little is known about how Skd3 mutations might cause disease.

Collectively, these various observations concerning Skd3 led us to hypothesize that Skd3 is a metazoan mitochondrial protein disaggregase of key importance for mitochondrial proteostasis. We further hypothesized that MGCA7-associated Skd3 mutations would disrupt disaggregase activity. Our investigation of these hypotheses is detailed below. Briefly, we find that Skd3 is an ATP-dependent mitochondrial protein disaggregase that is activated by the rhomboid protease, PARL, and inactivated by MGCA7-linked mutations.

## Results

### Skd3 couples ATP hydrolysis to protein disaggregation and reactivation

**Figure 2.**
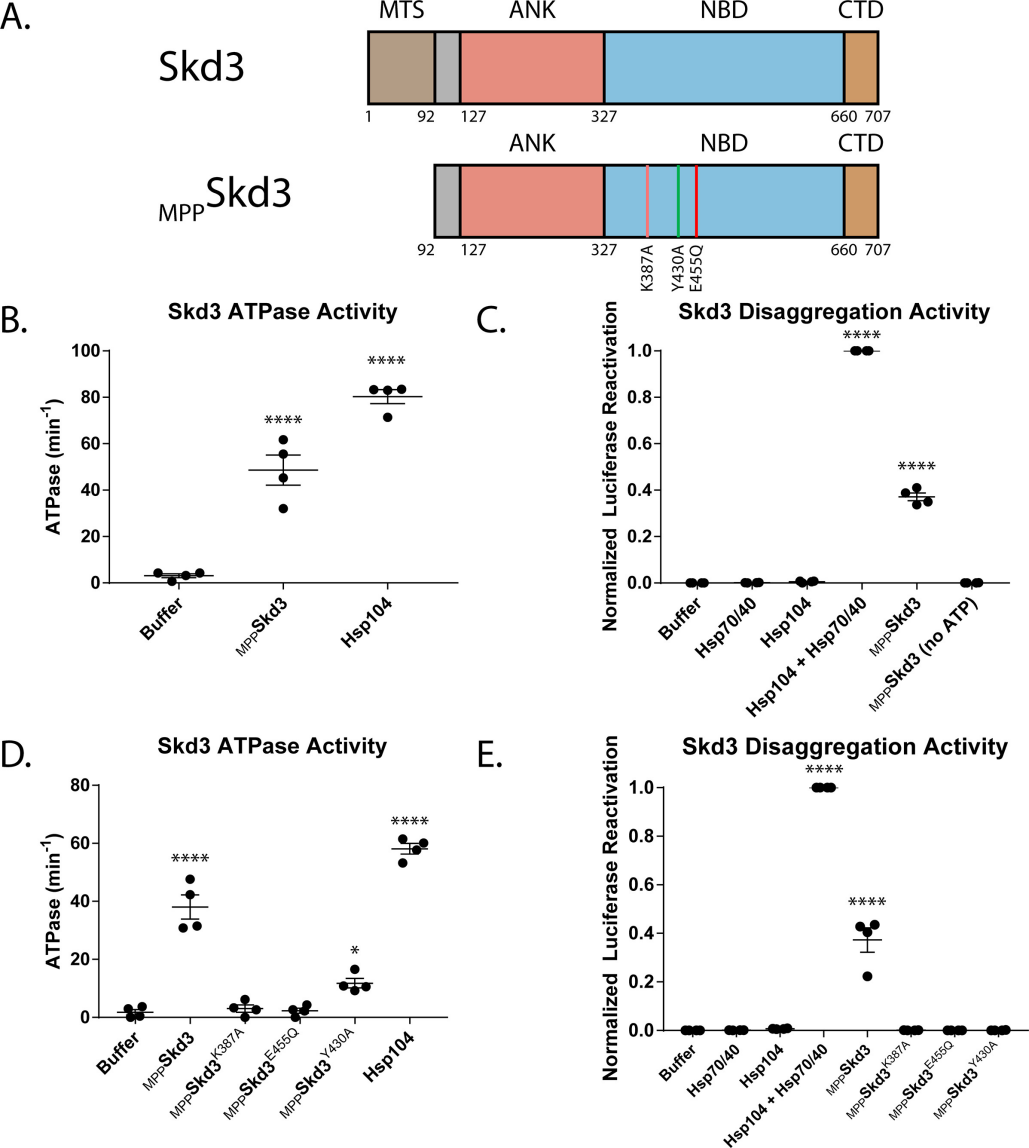
Skd3 is a protein disaggregase. (**A**) Domain map depicting the Mitochondrial-processing peptidase (MPP) cleavage site and mature-length Skd3 (_MPP_Skd3). The MTS was predicted using MitoProt in agreement with previous work, as outlined in the Materials and methods. The positions of the Walker A mutation (K387A) predicted to disrupt ATP binding and hydrolysis, pore-loop tyrosine mutation (Y430A) predicted to disrupt substrate binding, and Walker B mutation (E455Q) predicted to disrupt ATP hydrolysis are shown. (**B**) _MPP_Skd3 is an ATPase. ATPase assay comparing _MPP_Skd3 and Hsp104. _MPP_Skd3 and Hsp104 ATPase were compared to buffer using one-way ANOVA and a Dunnett’s multiple comparisons test (N = 4, individual data points shown as dots, bars show mean ± SEM, ****p<0.0001). (**C**) Luciferase disaggregation/reactivation assay showing that _MPP_Skd3 has disaggregase activity in the presence but not absence of ATP. Luciferase activity was buffer subtracted and normalized to Hsp104 + Hsp70/Hsp40. Luciferase activity was compared to buffer using one-way ANOVA and a Dunnett’s multiple comparisons test (N = 4, individual data points shown as dots, bars show mean ± SEM, ****p<0.0001). (**D**) ATPase assay comparing _MPP_Skd3, _MPP_Skd3^K387A^ (Walker A mutant), _MPP_Skd3^E455Q^ (Walker B mutant), and _MPP_Skd3^Y430A^ (Pore-Loop mutant), showing that both Walker A and Walker B mutations abolish Skd3 ATPase activity, whereas the Pore Loop mutation reduces ATPase activity. ATPase activity was compared to buffer using one-way ANOVA and a Dunnett’s multiple comparisons test (N = 4, individual data points shown as dots, bars show mean ± SEM, *p<0.05, ****p<0.0001). (**E**) Luciferase disaggregation/reactivation assay comparing _MPP_Skd3 to Walker A, Walker B, and Pore-Loop variants demonstrating that ATP binding, ATP hydrolysis, and pore-loop contacts are essential for Skd3 disaggregase activity. Luciferase activity was buffer subtracted and normalized to Hsp104 + Hsp70/Hsp40. Luciferase activity was compared to buffer using one-way ANOVA and a Dunnett’s multiple comparisons test (N = 4, individual data points shown as dots, bars show mean ± SEM, ****p<0.0001).

To biochemically dissect the activity of Skd3, we purified full-length Skd3 (see Materials and methods), lacking the mitochondrial targeting signal, which is cleaved by the mitochondrial-processing peptidase (MPP) upon import into mitochondria (Figure 2a, Figure 2—figure supplement 1a–d; Claros and Vincens, 1996; Wortmann et al., 2015). We term this form of Skd3, _MPP_Skd3. We first assessed that ATPase activity of _MPP_Skd3 and found that it is active (Figure 2b and Figure 2—figure supplement 2a). Indeed, _MPP_Skd3 displayed robust ATPase activity that was comparable to Hsp104 and over two-fold higher than previously reported values for _MPP_Skd3 activity (Figure 2b and Figure 2—figure supplement 2a; Mróz et al., 2020; Wortmann et al., 2015).

**Figure 2—figure supplement 1.**
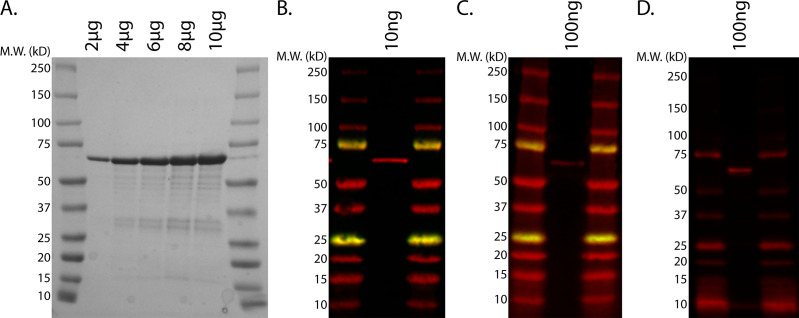
Recombinant Skd3 is highly pure and immunoreactive with several commercially available antibodies. (**A**) Representative gel of _MPP_Skd3 showing high purity via Coomassie Brilliant Blue stain. (**B**) Western blot with Skd3 antibody (Abcam ab76179) showing immunoreactivity of a singular band of purified _MPP_Skd3. (**C**) Western blot with Skd3 antibody (Abcam ab87253) showing immunoreactivity of a singular band of purified _MPP_Skd3. (**D**) Western blot with Skd3 antibody (Proteintech #15743–1-AP) showing immunoreactivity of a singular band of purified _MPP_Skd3.

**Figure 2—figure supplement 2.**
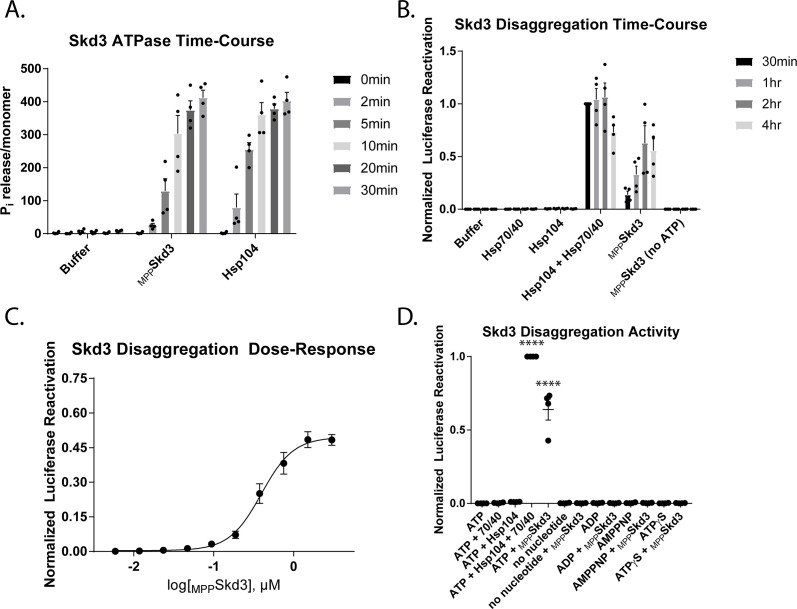
Skd3 is a protein disaggregase. (**A**) ATPase assay time course showing that _MPP_Skd3 ATPase activity is approximately linear over the first five minutes of the assay. (N = 4, bars show mean ± SEM). (**B**) Luciferase disaggregation/reactivation activity time course showing that _MPP_Skd3 disaggregates more luciferase over time. Luciferase activity was buffer subtracted and normalized to Hsp104 plus Hsp70 and Hsp40 (N = 4, bars show mean ± SEM). (**C**) Luciferase disaggregation/reactivation assay showing dose-response relationship between _MPP_Skd3 concentration and luciferase reactivation. Luciferase activity was buffer subtracted and normalized to Hsp104 plus Hsp70 and Hsp40. (N = 4, dots show mean ± SEM, EC_50_ = 0.394 μM). (**D**) Luciferase disaggregation/reactivation assay showing _MPP_Skd3 disaggregase activity in the presence of different nucleotides. Results show that _MPP_Skd3 can disaggregate luciferase in the presence of ATP, but not in the absence of ATP, in the presence of ADP, or in the presence of ATP analogues ATPγS (slowly hydrolyzable) or AMP-PNP (non-hydrolyzable). Luciferase assay incubated for 30 min and no ATP regeneration system was used. Luciferase activity was buffer subtracted and normalized to Hsp104 plus Hsp70 and Hsp40 (N = 4, individual data points shown as dots, bars show mean ± SEM, ****p<0.0001).

To determine if _MPP_Skd3 is a disaggregase we used a classic aggregated substrate, urea-denatured firefly luciferase aggregates, which form aggregated structures of ∼500–2000 kDa and greater in size that are devoid of luciferase activity (DeSantis et al., 2012; Glover and Lindquist, 1998). Indeed, very few luciferase species smaller than ~400 kDa can be detected (Glover and Lindquist, 1998). Importantly, these samples are devoid of misfolded, monomeric luciferase (Glover and Lindquist, 1998). Reactivation of luciferase in this assay is thus primarily achieved by the extraction and subsequent refolding of monomeric luciferase (M_w_ ~61 kDa) from large aggregated structures (∼500–2,000 kDa or larger) (Glover and Lindquist, 1998). Hence, in this assay, luciferase reactivation is an accurate proxy for luciferase disaggregation. Importantly, Hsp70 and Hsp40 are unable to disaggregate and reactivate luciferase found in these large aggregated structures (∼500–2,000 kDa or larger) (Glover and Lindquist, 1998). Indeed, Hsp70 and Hsp40 were unable to recover any active luciferase (~0.05 ± 0.00% [mean ± SEM] native luciferase activity) and were similar to the buffer control (~0.04 ± 0.00% native luciferase activity) (Figure 2c and Figure 2—figure supplement 2b,d). As expected, Hsp104 alone was also unable to disaggregate and reactivate luciferase (~0.08 ± 0.01% native luciferase activity) (Figure 2c and Figure 2—figure supplement 2b,d; DeSantis et al., 2012; Glover and Lindquist, 1998). By contrast, the combination of Hsp104, Hsp70, and Hsp40 recovered ~7.68 ± 0.44% native luciferase activity (Figure 2c and Figure 2—figure supplement 2b,d; Glover and Lindquist, 1998). Remarkably, _MPP_Skd3 displayed robust disaggregase activity in the presence of ATP as it was able to recover ~2.87 ± 0.17% native luciferase activity (Figure 2c and Figure 2—figure supplement 2b,c). Indeed, _MPP_Skd3 displayed ~40% of the disaggregase activity of Hsp104 plus Hsp70 and Hsp40 under these conditions (Figure 2c). While Hsp104 required the presence of Hsp70 and Hsp40 to disaggregate luciferase (Figure 2c and Figure 2—figure supplement 2b,c; DeSantis et al., 2012; Glover and Lindquist, 1998), _MPP_Skd3 had no requirement for Hsp70 and Hsp40 (Figure 2c and Figure 2—figure supplement 2b,c). This finding indicates that _MPP_Skd3 is a ‘stand-alone’ disaggregase.

Next, we assessed the nucleotide requirements for _MPP_Skd3 disaggregase activity. _MPP_Skd3 disaggregase activity was supported by ATP but not by the absence of nucleotide or the presence of ADP (Figure 2c and Figure 2—figure supplement 2b,d). Likewise, neither the non-hydrolyzable ATP analogue, AMP-PNP, nor the slowly hydrolyzable ATP analogue, ATPγS, could support _MPP_Skd3 disaggregase activity (Figure 2—figure supplement 2b,d). Collectively, these data suggest that _MPP_Skd3 disaggregase activity requires multiple rounds of rapid ATP binding and hydrolysis, which is similar mechanistically to Hsp104 (DeSantis et al., 2012; Shorter and Lindquist, 2004; Shorter and Lindquist, 2006).

We next investigated the role of conserved AAA+ elements in Skd3 activity. Thus, we mutated: (1) a critical lysine in the Walker A motif to alanine (K387A), which is predicted to disrupt ATP binding and hydrolysis (Hanson and Whiteheart, 2005; Seraphim and Houry, 2020); (2) a critical glutamate in the Walker B motif to glutamine (E455Q), which is predicted to disrupt ATP hydrolysis but not ATP binding (Hanson and Whiteheart, 2005; Seraphim and Houry, 2020); and (3) a highly-conserved tyrosine in the predicted -GYVG- substrate-binding loop to alanine that is predicted to disrupt substrate binding (Y430A) as in related HCLR clade AAA+ ATPases (Gates et al., 2017; Hanson and Whiteheart, 2005; Lopez et al., 2020; Martin et al., 2008; Rizo et al., 2019; Seraphim and Houry, 2020). The equivalent Walker A, Walker B, and substrate-binding loop mutations in NBD1 and NBD2 of Hsp104 severely disrupt disaggregase activity (DeSantis et al., 2012; Lum et al., 2004; Torrente et al., 2016). Likewise, _MPP_Skd3^K387A^ (Walker A mutant) and _MPP_Skd3^E455Q^ (Walker B mutant) displayed greatly reduced ATPase and disaggregase activity (Figure 2d,e). Thus, _MPP_Skd3 couples ATP binding and hydrolysis to protein disaggregation.

Interestingly, the pore-loop variant, _MPP_Skd3^Y430A^, exhibited reduced ATPase activity compared to _MPP_Skd3, but much higher ATPase activity than _MPP_Skd3^K387A^ and _MPP_Skd3^E455Q^ (Figure 2d). This reduction in ATPase activity was unexpected as equivalent mutations in Hsp104 do not affect ATPase activity (DeSantis et al., 2012; Lum et al., 2008; Lum et al., 2004; Torrente et al., 2016). _MPP_Skd3^Y430A^ was also devoid of disaggregase activity (Figure 2e). The inhibition of disaggregase activity by Y430A was much more severe than the inhibition of ATPase activity (Figure 2d,e), which suggests that the pore-loop Y430 might make direct contact with substrate to drive protein disaggregation as in Hsp104 (DeSantis et al., 2012; Gates et al., 2017). Thus, the conserved putative substrate-binding tyrosine of the -GYVG- pore-loop is critical for _MPP_Skd3 disaggregase activity.

### Skd3 disaggregase activity is enhanced by PARL cleavage

We noticed that Skd3 contains an undefined, 35-amino acid, hydrophobic stretch between the N-terminal MTS and the ankyrin-repeat domain (Figure 1a and Figure 3—figure supplement 1). Intriguingly, Skd3 is cleaved by the inner-membrane rhomboid protease, PARL, at amino acid 127, between the 35-amino acid, hydrophobic stretch and the ankyrin-repeat domain (Figure 3—figure supplement 1; Saita et al., 2017; Spinazzi et al., 2019). Sequence analysis shows that the 35-amino acid, hydrophobic stretch and the PARL-cleavage motif are both highly conserved among mammalian Skd3 orthologues (Figure 3—figure supplement 2a). Thus, we hypothesized that this 35-amino acid, hydrophobic stretch might be auto-inhibitory for Skd3 activity.

**Figure 3.**
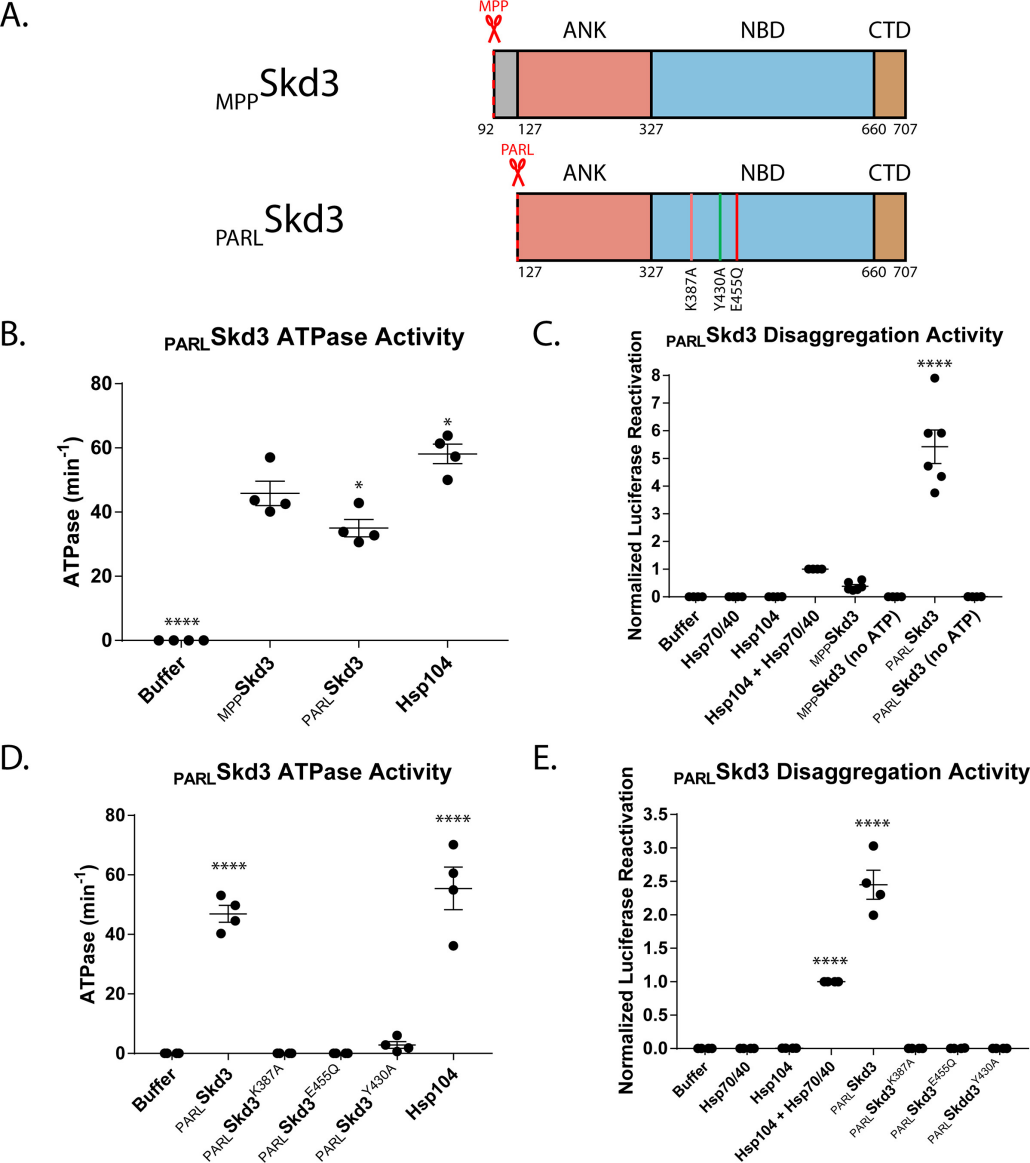
PARL cleavage enhances Skd3 disaggregase activity. (**A**) Domain map depicting _MPP_Skd3 and the PARL cleavage site and corresponding PARL-cleaved Skd3 (_PARL_Skd3). The positions of the Walker A mutation (K387A) predicted to disrupt ATP binding and hydrolysis, pore-loop tyrosine mutation (Y430A) predicted to disrupt substrate binding, and Walker B mutation (E455Q) predicted to disrupt ATP hydrolysis are shown. (**B**) ATPase assay comparing _MPP_Skd3 and _PARL_Skd3. _PARL_Skd3 is catalytically active, but is slightly less active than _MPP_Skd3. _PARL_Skd3 and Hsp104 ATPase were compared to _MPP_Skd3 ATPase using one-way ANOVA and a Dunnett’s multiple comparisons test (N = 6, individual data points shown as dots, bars show mean ± SEM, *p<0.05, ****p<0.0001). (**C**) Luciferase disaggregation/reactivation assay comparing _MPP_Skd3 disaggregase activity to _PARL_Skd3. _PARL_Skd3 was over 10-fold more active than _MPP_Skd3. Luciferase activity was buffer subtracted and normalized to Hsp104 + Hsp70/Hsp40. Luciferase activity was compared to _MPP_Skd3 using one-way ANOVA and a Dunnett’s multiple comparisons test (N = 6, individual data points shown as dots, bars show mean ± SEM, ****p<0.0001). (**D**) ATPase assay comparing _PARL_Skd3, _PARL_Skd3^K387A^ (Walker A), _PARL_Skd3^E455Q^ (Walker B), and _PARL_Skd3^Y430A^ (Pore Loop), showing that both Walker A and Walker B mutations abolish Skd3 ATPase activity, whereas the Pore-Loop mutation reduces ATPase activity. ATPase activity was compared to buffer using one-way ANOVA and a Dunnett’s multiple comparisons test (N = 4, individual data points shown as dots, bars show mean ± SEM, ****p<0.0001). (**E**) Luciferase disaggregation/reactivation assay comparing _PARL_Skd3 to Walker A, Walker B, and Pore-Loop variants showing that ATP binding, ATP hydrolysis, and pore-loop contacts are essential for _PARL_Skd3 disaggregase activity. Luciferase activity was buffer subtracted and normalized to Hsp104 + Hsp70/Hsp40. Luciferase activity was compared to buffer using one-way ANOVA and a Dunnett’s multiple comparisons test (N = 4, individual data points shown as dots, bars show mean ± SEM, ****p<0.0001).

To determine whether PARL cleavage of this 35-amino acid, hydrophobic stretch regulates Skd3 activity, we purified Skd3 without this region (_PARL_Skd3) (Figure 3a). We found that the absence of the 35-residue stretch that is removed by PARL slightly decreased Skd3 ATPase activity compared to _MPP_Skd3 (Figure 3b and Figure 3—figure supplement 2b). Moreover, we find that _MPP_Skd3 ATPase activity is not stimulated by the model substrate, casein, a classic peptide-stimulator of Hsp104 ATPase activity (Figure 3—figure supplement 3; Cashikar et al., 2002; Gates et al., 2017). By contrast, _PARL_Skd3 ATPase activity is mildly stimulated by casein (Figure 3—figure supplement 3). This finding indicates that _PARL_Skd3 may interact more effectively with substrates than _MPP_Skd3 due to the removal of the 35-amino acid, hydrophobic stretch.

**Figure 3—figure supplement 1.**
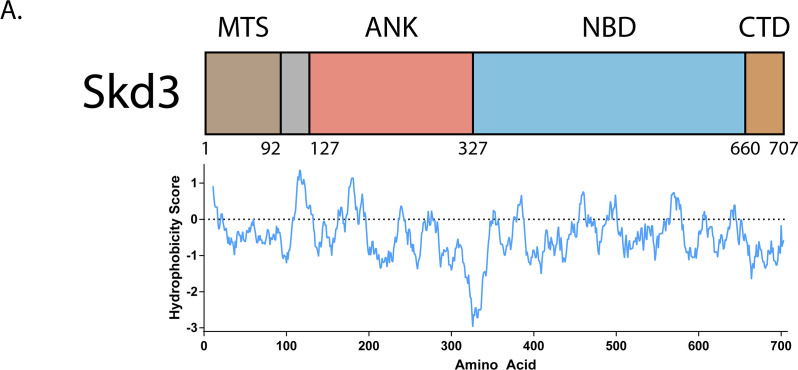
The auto-inhibitory peptide of Skd3 is hydrophobic. (**A**) Kyte-Doolittle hydrophobicity score was calculated for Skd3 using the ExPASy web server (Kyte and Doolittle, 1982; Wilkins et al., 1999). A positive hydrophobicity score indicates highly hydrophobic regions. Analysis shows a spike in hydrophobicity corresponding to the C-terminal portion of the Skd3 auto-inhibitory peptide (92-126), which is removed by PARL cleavage.

**Figure 3—figure supplement 2.**
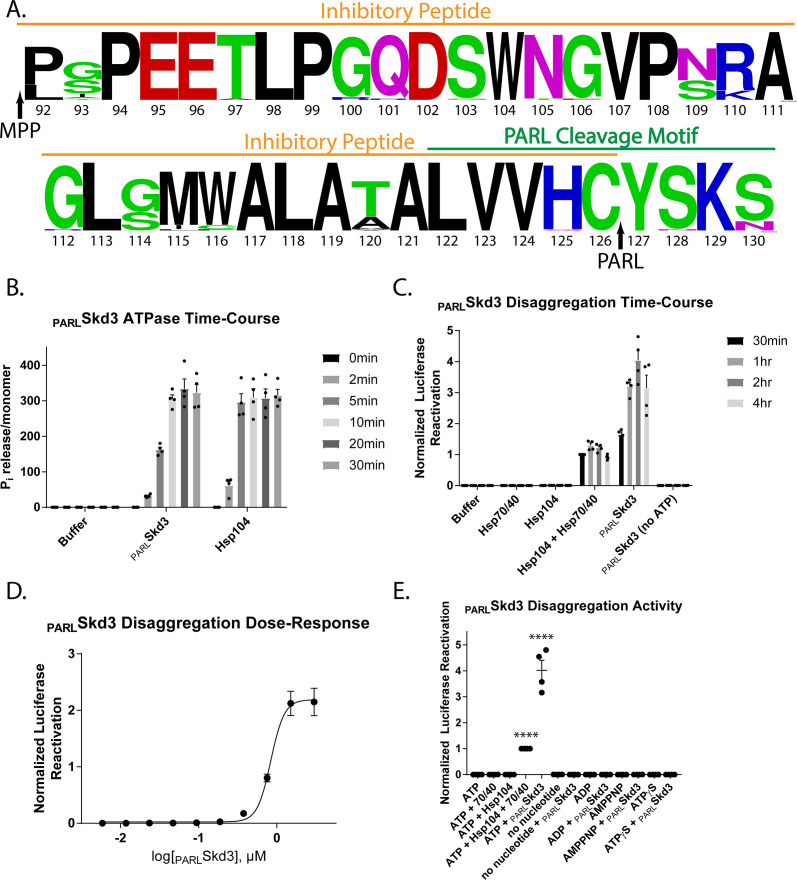
PARL cleavage of Skd3 enhances Skd3 disaggregase activity. (**A**) Sequence logo depicting the conservation of the auto-inhibitory peptide (orange) and PARL-cleavage motif (green) of Skd3. Arrows indicate MPP and PARL cleavage sites. Logo shows a high level of homology suggesting conserved importance. Sequence Logo was built with WebLogo using Skd3 protein sequence from 42 different mammalian species. (**B**) ATPase assay time course showing that _PARL_Skd3 ATPase activity is approximately linear over the first five minutes of the assay (N = 4, bars show mean ± SEM). (**C**) Luciferase disaggregation/reactivation activity time course showing that _PARL_Skd3 disaggregates more luciferase over time. Luciferase activity was buffer subtracted and normalized to Hsp104 plus Hsp70 and Hsp40 (N = 4, bars show mean ± SEM). (**D**) Luciferase disaggregation/reactivation assay showing dose-response relationship between _PARL_Skd3 concentration and luciferase reactivation. Luciferase activity was buffer subtracted and normalized to Hsp104 plus Hsp70 and Hsp40 (N = 4, dots show mean ± SEM, EC_50_ = 0.836 μM). (**E**) Luciferase disaggregation/reactivation assay showing _PARL_Skd3 disaggregase activity in the presence of different nucleotides. Results show that _PARL_Skd3 can disaggregate luciferase in the presence of ATP, but not in the absence of ATP, in the presence of ADP, or in the presence of ATP analogues ATPγS (slowly hydrolyzable) or AMP-PNP (non-hydrolyzable). Luciferase assay incubated for 30 min and no ATP regeneration system was used. Luciferase activity was buffer subtracted and normalized to Hsp104 plus Hsp70 and Hsp40 (N = 4, individual data points shown as dots, bars show mean ± SEM, ****p<0.0001).

**Figure 3—figure supplement 3.**
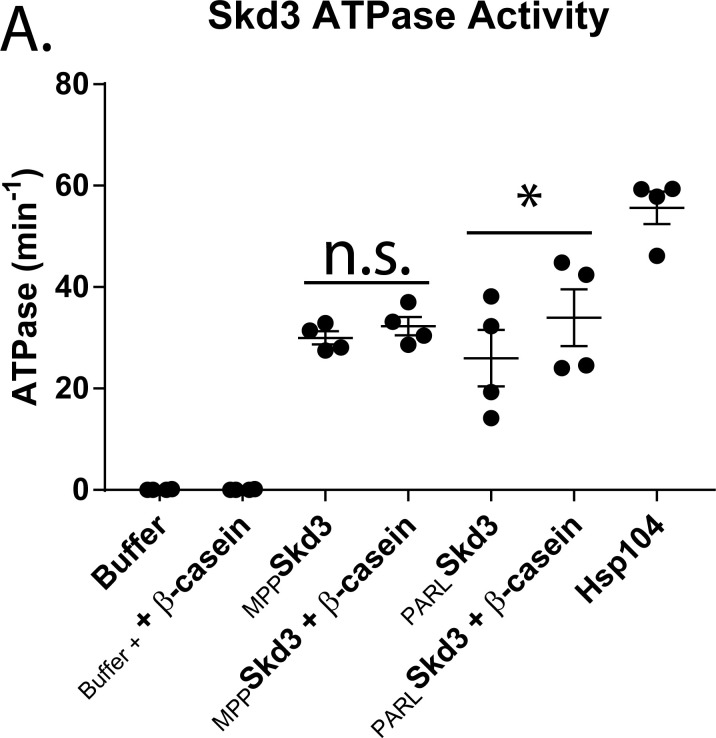
_PARL_Skd3, but not _MPP_Skd3, ATPase activity is stimulated by a model substrate. (**A**) ATPase assay showing that _PARL_Skd3 but not _MPP_Skd3 is stimulated by the model substrate β-casein. ATPase activity with substrate was compared to controls without substrate using a two-tailed, unpaired t-test. (N = 4, individual data points shown as dots, bars show mean ± SEM, *p<0.05).

Remarkably, the absence of the N-terminal peptide known to be removed by PARL unleashed Skd3 disaggregase activity (Figure 3c and Figure 3—figure supplement 2c,d). Indeed, _PARL_Skd3 exhibited over 10-fold higher disaggregase activity (~44.06 ± 3.78% native luciferase activity recovered, mean ± SEM) compared to _MPP_Skd3 (~3.10 ± 0.44% native luciferase activity recovered) and over five-fold higher disaggregation activity than Hsp104 plus Hsp70 and Hsp40 (~8.58 ± 1.20% native luciferase activity recovered), despite _PARL_Skd3 having lower ATPase activity when compared to _MPP_Skd3 (Figure 3b,c, and Figure 3—figure supplement 2b,c,d). These results suggest that Skd3 disaggregase activity is likely regulated by PARL and that PARL-activated Skd3 is a powerful, stand-alone protein disaggregase with activity comparable to potentiated Hsp104 variants (Jackrel et al., 2014; Jackrel and Shorter, 2014; Jackrel et al., 2015; Tariq et al., 2019; Tariq et al., 2018; Torrente et al., 2016).

As with _MPP_Skd3, we found that _PARL_Skd3 disaggregase activity was supported by ATP, but not in the absence of nucleotide or in the presence of ADP, non-hydrolyzable AMP-PNP, or slowly hydrolyzable ATPγS (Figure 3c and Figure 3—figure supplement 2e). Likewise, _PARL_Skd3^K387A^ (Walker A mutant) and _PARL_Skd3^E455Q^ (Walker B mutant) lacked ATPase and disaggregase activity (Figure 3d,e), indicating that _PARL_Skd3 couples ATP binding and hydrolysis to protein disaggregation. Curiously, _PARL_Skd3^Y430A^ (pore-loop mutant) exhibited a larger reduction in ATPase activity than _MPP_Skd3^Y430A^ (Figures 2d and 3d), indicating that the conserved tyrosine in the conserved putative substrate-binding -GYVG- pore loop impacts ATPase activity in Skd3, whereas it has no effect in Hsp104 (DeSantis et al., 2012; Torrente et al., 2016). _PARL_Skd3^Y430A^ was devoid of disaggregase activity (Figure 3e), which could be due to reduced ATPase activity, reduced substrate binding, or both.

### PARL-activated Skd3 solubilizes α-synuclein fibrils

**Figure 4.**
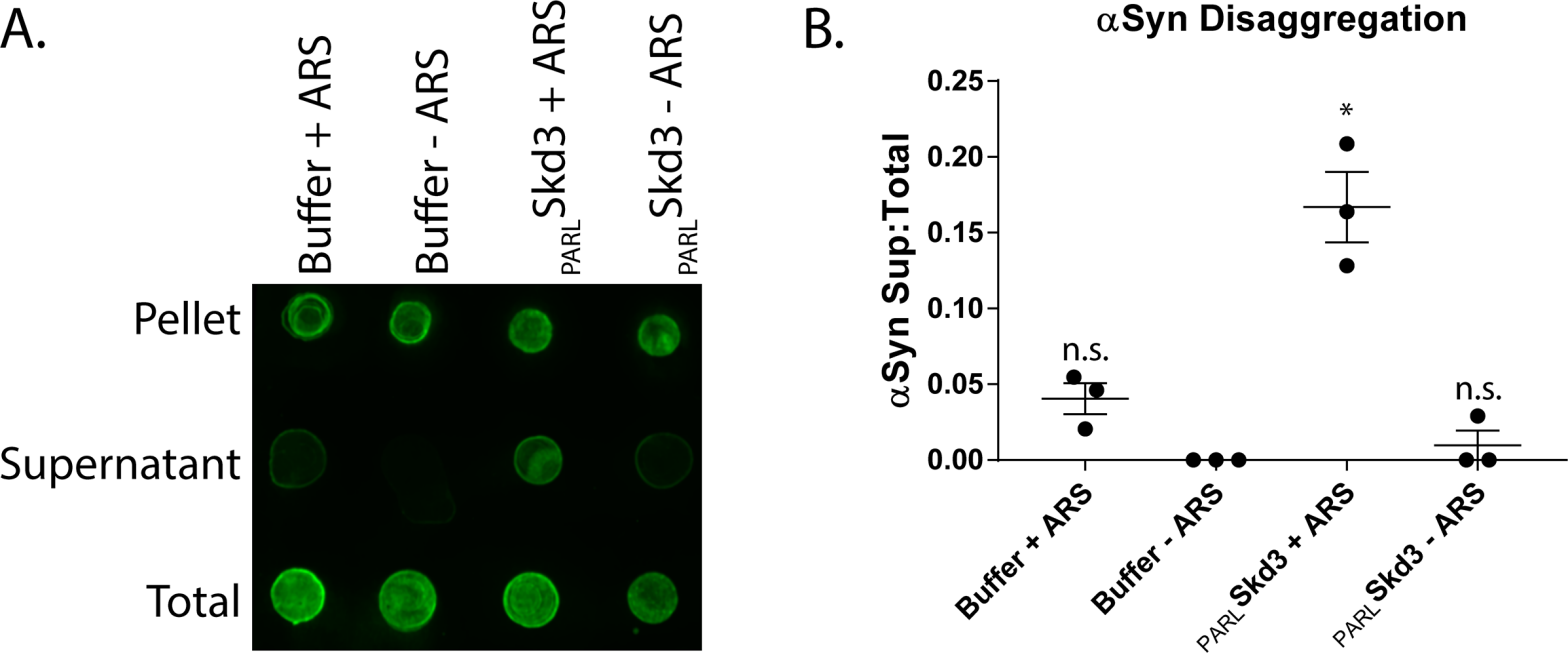
Skd3 disaggregates α-synuclein fibrils. (**A**) Representative dot blot of α-synuclein disaggregation assay. Blot shows solubilization of α-synuclein fibrils by _PARL_Skd3 in the presence of an ATP regeneration system (ARS), but not in the presence of _PARL_Skd3 or ARS alone. (N = 3). (**B**) Quantification of α-synuclein disaggregation assay showing that _PARL_Skd3 in the presence of an ARS disaggregates α-synuclein fibrils. Results are normalized as fraction in the supernatant relative to the fraction in the supernatant and the pellet. The fraction of α-synuclein in the supernatant was compared to buffer using a repeated measure one-way ANOVA and a Dunnett’s multiple comparisons test (N = 3, individual data points shown as dots, bars show mean ± SEM, *p<0.05).

Next, we assessed whether _PARL_Skd3 could disassemble a stable amyloid substrate, which makes more stringent demands on a disaggregase (DeSantis et al., 2012). Thus, we turned to α-synuclein fibrils, which are connected to Parkinson’s disease and various synucleinopathies (Henderson et al., 2019; Spillantini et al., 1998a; Spillantini et al., 1998b). We utilized a strain of synthetic α-synuclein fibrils capable of eliciting Parkinson’s disease-like symptoms in mice (Luk et al., 2012). We employed samples of preformed α-synuclein fibrils that had completely assembled (i.e. 100% of α-synuclein was in the assembled fibril state) and were devoid of soluble α-synuclein (Figure 4a,b). Using a sedimentation assay combined with a dot-blot, we found that _PARL_Skd3, but not _MPP_Skd3 (data not shown), disaggregated these disease-causing fibrils in the presence, but not absence of ATP (Figure 4a,b). Thus, _PARL_Skd3 is a powerful protein disaggregase, which could have applications as a potential therapeutic agent to eliminate disease-causing α-synuclein fibrils that underlie Parkinson’s disease and other synucleinopathies.

### Skd3 disaggregase activity requires the ankyrin-repeat domain and NBD

**Figure 5.**
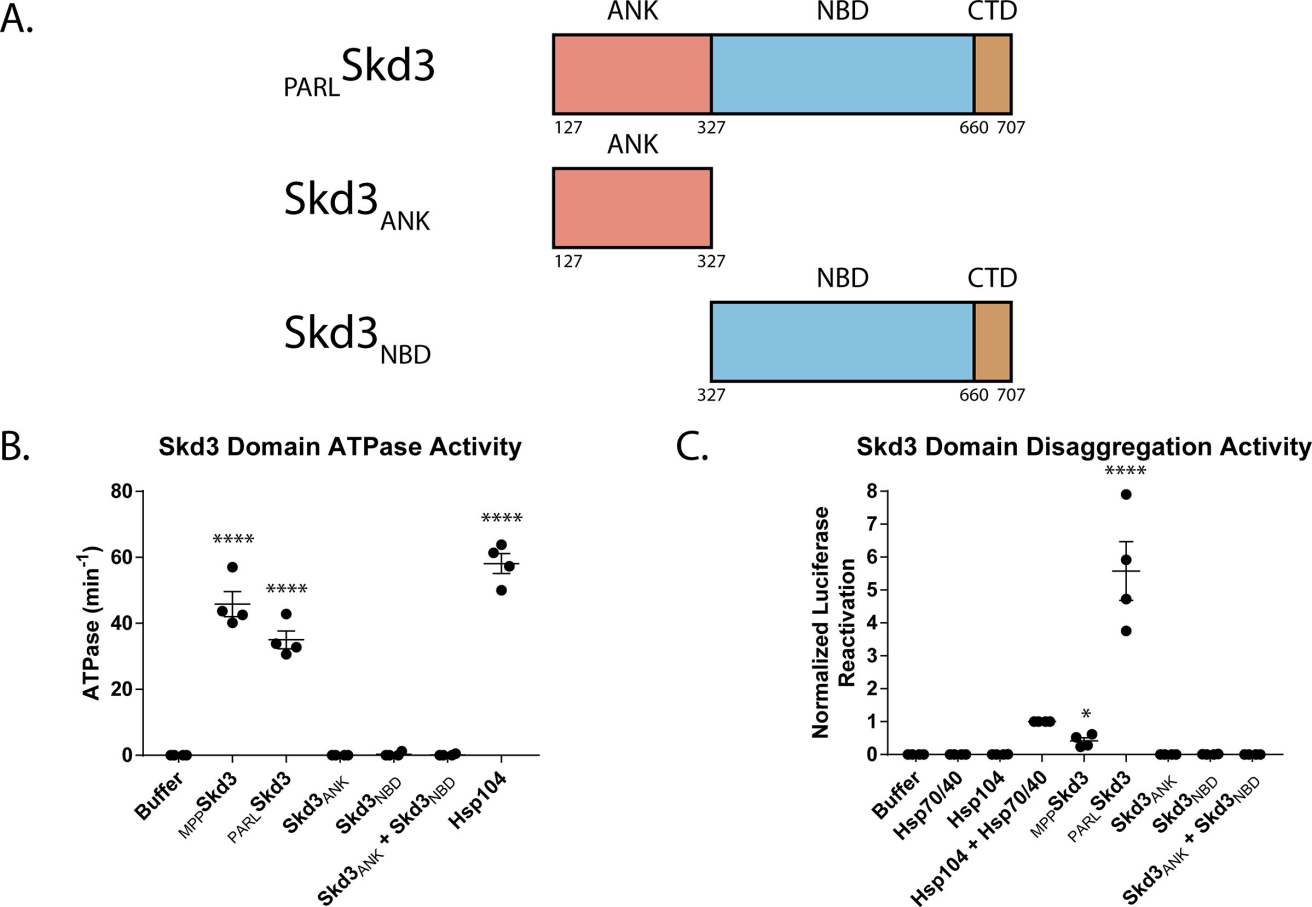
The ankyrin-repeat domain and NBD are required for Skd3 disaggregase activity. (**A**) Domain maps showing the Skd3_ANK_ and Skd3_NBD_ constructs. (**B**) ATPase assay comparing Skd3_ANK_ and Skd3_NBD_ ATPase activity. Results show that Skd3_ANK_, Skd3_NBD_, and Skd3_ANK_ + Skd3_NBD_ do not have ATPase activity. Data are from the same experiments as Figure 3B. ATPase activity was compared to buffer using one-way ANOVA and a Dunnett’s multiple comparisons test (N = 4, individual data points shown as dots, bars show mean ± SEM, ****p<0.0001). (**C**) Luciferase disaggregation/reactivation assay comparing Skd3_ANK_, Skd3_NBD_, and Skd3_ANK_ + Skd3_NBD_ d activity. Results show that Skd3_ANK_, Skd3_NBD_, or Skd3_ANK_ + Skd3_NBD_ are inactive disaggregases. Data are from same experiments as Figure 3C. Luciferase activity was buffer subtracted and normalized to Hsp104 plus Hsp70 and Hsp40. Luciferase disaggregase activity was compared to buffer using one-way ANOVA and a Dunnett’s multiple comparisons test (N = 4, individual data points shown as dots, bars show mean ± SEM, ****p<0.0001).

To further investigate the mechanism of Skd3 disaggregase activity, we purified the isolated ankyrin-repeat domain and NBD as separate proteins (Figure 5a). Neither the isolated ankyrin-repeat domain nor the isolated NBD exhibited ATPase activity or disaggregase activity (Figure 5b,c). The lack of ATPase activity and disaggregase activity of the isolated NBD is consistent with a similar lack of activity of isolated NBD2 from Hsp104 or bacterial ClpB and is likely attributed to a lack of hexamer formation, as is observed for ClpB NBD2 (Beinker et al., 2005; Hattendorf and Lindquist, 2002; Mogk et al., 2003). Thus, the ankyrin-repeat domain and NBD combine in *cis* to enable Skd3 ATPase activity and disaggregase activity. We also tested whether the two domains could combine in *trans* as two separate proteins to yield an active ATPase or disaggregase. However, we found that equimolar amounts of the ankyrin-repeat domain and NBD were also inactive (Figure 5b,c). Thus, Skd3 is unlike bacterial ClpB, which can be reconstituted in *trans* by separate NTD-NBD1-MD and NBD2 proteins (Beinker et al., 2005). These findings suggest that the covalent linkage of the ankyrin-repeat domain and NBD is critical for forming a functional ATPase and disaggregase. Importantly, they also predict that truncated MGCA7-linked Skd3 variants, such as R250* and K321* (where * indicates a stop codon), which lack the NBD would be inactive for protein disaggregation and indeed any ATPase-dependent activities.

### Skd3 disaggregase activity is not stimulated by Hsp70 and Hsp40

**Figure 6.**
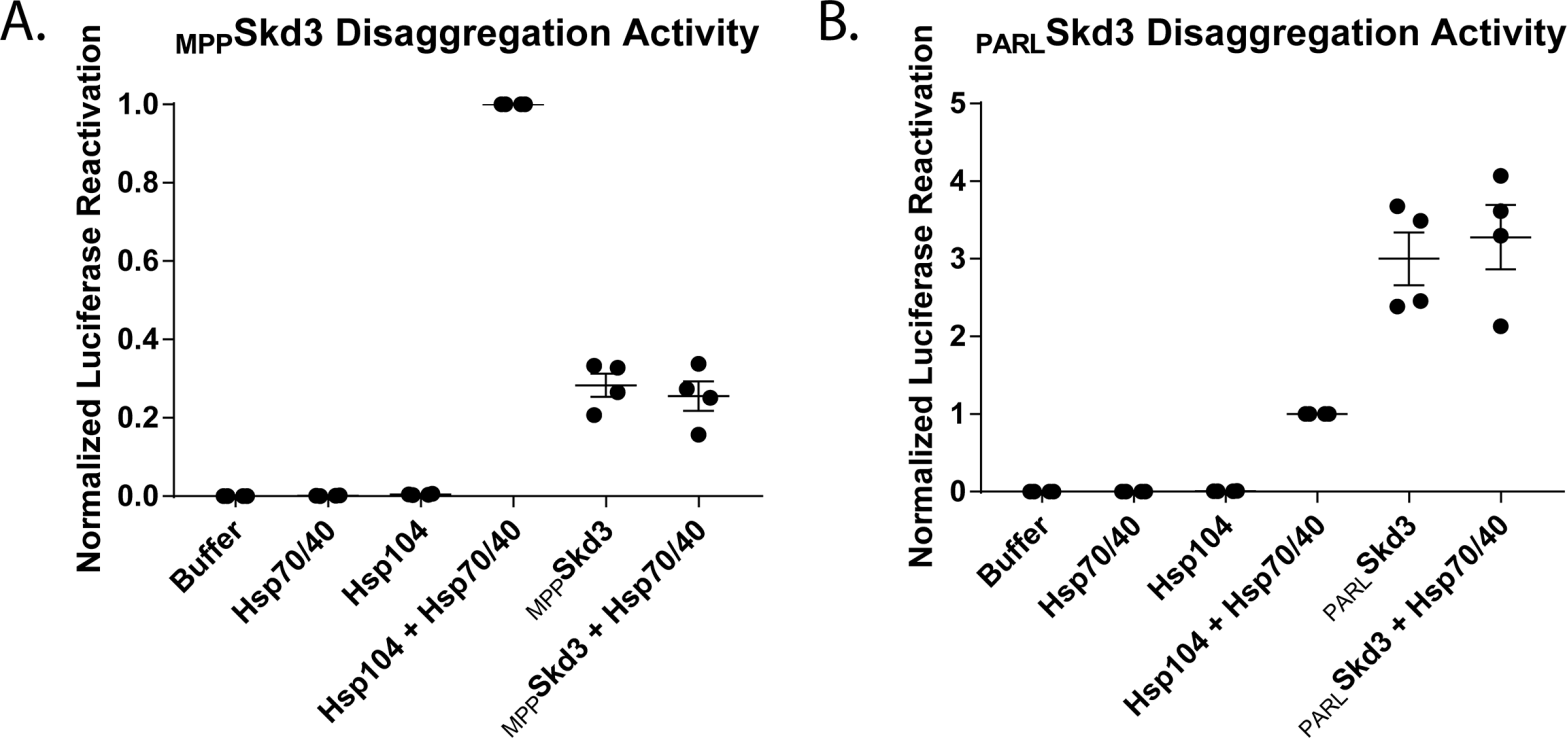
Skd3 does not collaborate with Hsp70 and Hsp40 in protein disaggregation. (**A**) Luciferase disaggregation/reactivation comparing _MPP_Skd3 disaggregase activity in the presence or absence of Hsp70 (Hsc70) and Hsp40 (Hdj1). Luciferase activity was buffer subtracted and normalized to Hsp104 plus Hsp70 and Hsp40. Results show a stimulation of Hsp104 disaggregase activity by Hsp70 and Hsp40, but no stimulation of disaggregase activity for _MPP_Skd3. _MPP_Skd3 plus Hsp70 and Hsp40 was compared to _MPP_Skd3 using a two-tailed, unpaired t-test. Test found no significant difference in disaggregation activity. (N = 4, individual data points shown as dots, bars show mean ± SEM). (**B**) Luciferase disaggregation/reactivation comparing _PARL_Skd3 disaggregase activity in the presence or absence of Hsp70 and Hsp40. Luciferase activity was buffer subtracted and normalized to Hsp104 plus Hsp70 and Hsp40. Results show no stimulation of disaggregase activity for _PARL_Skd3 by Hsp70 and Hsp40. _PARL_Skd3 plus Hsp70 and Hsp40 was compared to _PARL_Skd3 using a two-tailed, unpaired t-test. Test found no significant difference in disaggregation activity. (N = 4, individual data points shown as dots, bars show mean ± SEM).

Hsp104 and Hsp78 collaborate with Hsp70 and Hsp40 to disaggregate many substrates (Figures 2c,e and 3c,e; DeSantis et al., 2012; Glover and Lindquist, 1998; Krzewska et al., 2001). By contrast, Skd3 does not require Hsp70 and Hsp40 for protein disaggregation (Figures 2c,e, 3c,e and 4a,b). This finding is consistent with Skd3 lacking the NTD, NBD1, and MD of Hsp104, which interact with Hsp70 (DeSantis et al., 2014; Lee et al., 2013; Sweeny et al., 2015; Sweeny et al., 2020). Nonetheless, Hsp70 and Hsp40 might still augment Skd3 disaggregase activity. Thus, we tested if Hsp70 and Hsp40 could stimulate Skd3 disaggregase activity. However, neither _MPP_Skd3 nor _PARL_Skd3disaggregase activity was stimulated by Hsp70 and Hsp40 (Figure 6a,b). Thus, Skd3 is a stand-alone disaggregase that works independently of the Hsp70 chaperone system.

### Human cells lacking Skd3 exhibit reduced solubility of mitochondrial proteins

**Figure 7.**
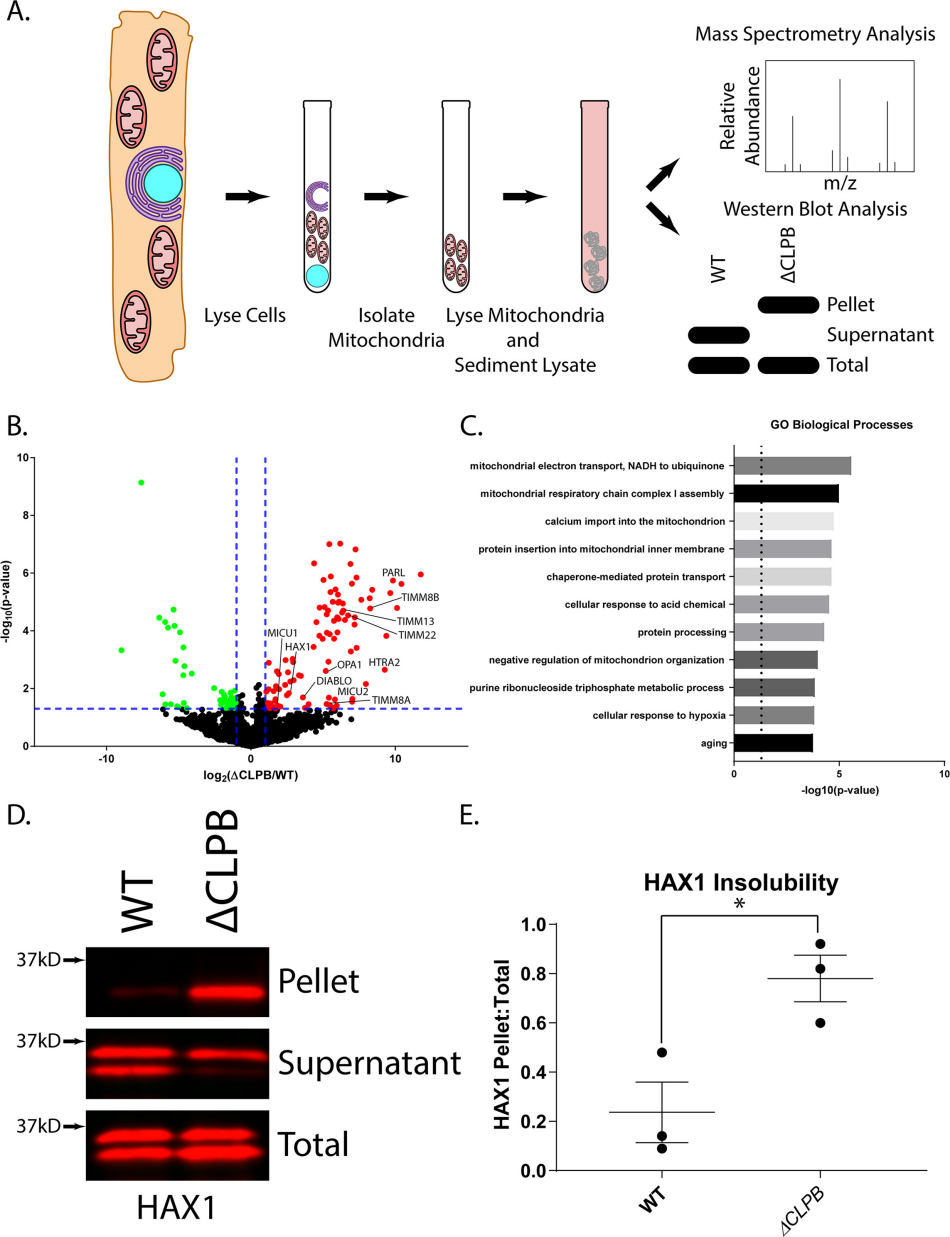
Skd3 maintains the solubility of key mitochondrial proteins in human cells. (**A**) Schematic showing sedimentation assay design. HAP1 cells were lysed and the mitochondrial fraction was separated from the cytosolic fraction. The mitochondrial fraction was then lysed, and the soluble fraction was separated from the insoluble fraction via sedimentation. The samples were then either analyzed via mass-spectrometry or western blotting. (**B**) Volcano plot showing the log_2_ fold change of protein in the Skd3 (*CLPB*) knockout pellet compared to the wild-type (WT) pellet. The 99 proteins that were enriched in the Skd3 pellet are highlighted in red. The 53 proteins that were enriched in the wild-type (WT) pellet are highlighted in green. Significance cutoffs were set as fold change >2.0 and p<0.05, indicated with blue dashed lines (N = 3, p<0.05). (**C**) Select statistically significant terms for GO biological processes from the enriched proteins in the Skd3 knockout pellet. Dashed line shows p=0.05 (p<0.05). For full list see Figure 7—figure supplement 2b. (**D**) Representative western blot of sedimentation assay showing relative solubility of HAX1 protein in wild-type (WT) and Skd3 (*CLPB*) knockout cells. Results show a marked decrease in HAX1 solubility when Skd3 is knocked out. (N = 3). (**E**) Quantification of HAX1 sedimentation assay shows an overall increase in the insoluble HAX1 relative to the total protein in the Skd3 (*CLPB*) knockout cell line. Quantification is normalized as signal in the pellet divided by the sum of the signal in the pellet and supernatant. The fraction in the pellet for the Skd3 knockout was compared to the wild-type cells using a two-way, unpaired, t-test. (N = 3, individual data points shown as dots, bars show mean ± SEM, *p<0.05).

Given the potent disaggregase activity of Skd3, we predicted that deletion of Skd3 in human cells would result in decreased protein solubility in mitochondria. To determine the effect of Skd3 on protein solubility in mitochondria, we compared the relative solubility of the mitochondrial proteome in wild-type and Skd3 knockout human HAP1 cells (Figure 7—figure supplement 1a–c) using mass spectrometry as described in Figure 7a. Overall, we observed decreased protein solubility in mitochondria from the Skd3 knockout cells when compared to their wild-type counterparts (Figure 7b and Figure 7—figure supplement 2a). Using Gene Ontology (GO) analysis for cellular component, we found that proteins in the inner mitochondrial membrane and intermembrane space were enriched in the insoluble fraction in the absence of Skd3 (Figure 7—figure supplement 2a; Ashburner et al., 2000; Mi et al., 2019; The Gene Ontology Consortium, 2019). Importantly, Skd3 has been localized to the mitochondrial intermembrane space (Botham et al., 2019; Hung et al., 2014; Yoshinaka et al., 2019). Using GO analysis for biological process, we found that proteins involved in calcium import into mitochondria, chaperone-mediated protein transport, protein insertion into the mitochondrial inner membrane, mitochondrial electron transport, mitochondrial respiratory-chain complex assembly, and cellular response to hypoxia are more insoluble in Skd3 knockout cells compared to wild-type cells (Figure 7c and Figure 7—figure supplement 2b; Ashburner et al., 2000; Mi et al., 2019; The Gene Ontology Consortium, 2019).

**Figure 7—figure supplement 1.**
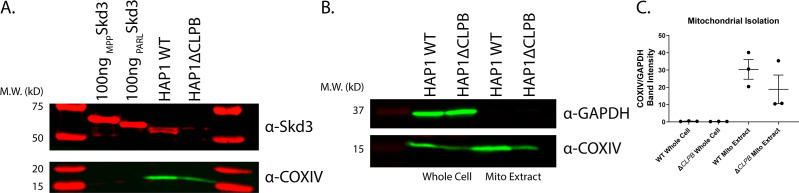
Verification of Skd3 knockout and mitochondria isolation in HAP1 cells. (**A**) Representative western blot of HAP1 cells showing knockout of Skd3. First and second lane show 100 ng load of recombinant _MPP_Skd3 and _PARL_Skd3. Anti-Skd3 (Abcam CAT# ab235349) and anti-COXIV (Abcam CAT# ab14744) antibodies were used. (**B**) Representative western blot of HAP1 whole cell lysate and mitochondria extract showing enrichment of the mitochondrial protein COXIV and depletion of the cytoplasmic protein GAPDH from whole cell lysates to mitochondrial extract (N = 3). (**C**) Quantification of the western blots from (**B**) showing the relative enrichment of COXIV:GAPDH in the purified mitochondria.

**Figure 7—figure supplement 2.**
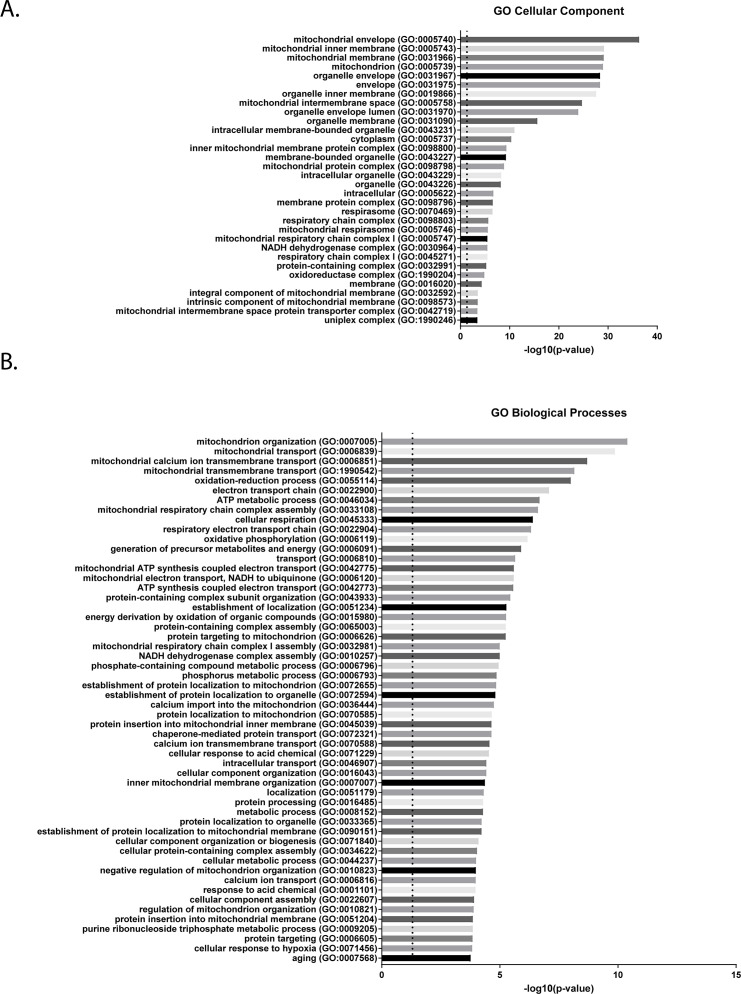
Skd3 deletion increases insolubility of mitochondrial inner membrane and intermembrane space proteins. (**A**) Terms for GO cellular component associated with the enriched proteins in the Skd3 knockout pellet. Dashed line shows p=0.05 (p<0.05). (**B**) Full list of terms for GO biological processes associated with the enriched proteins in the Skd3 knockout pellet. Dashed line shows p=0.05 (p<0.05).

**Figure 7—figure supplement 3.**
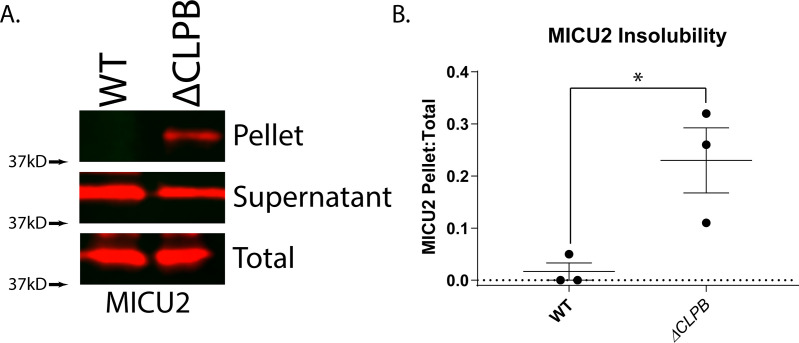
Skd3 maintains the solubility of MICU2 in human cells. (**A**) Representative western blot of sedimentation assay showing relative solubility of MICU2 protein in wild-type and Skd3 (*CLPB*) knockout cells. Results show a decrease in MICU2 solubility when Skd3 is knocked out. (N = 3). (**B**) Quantification of MICU2 sedimentation assay shows an overall increase in the insoluble MICU2 relative to the total protein in the Skd3 (*CLPB*) knockout cell line. Quantification is normalized as signal in the pellet divided by the sum of the signal in the pellet and supernatant. The fraction in the pellet for the Skd3 knockout was compared to the wild-type cells using a two-way, unpaired, t-test. (N = 3, individual data points shown as dots, bars show mean ± SEM, *p<0.05).

**Figure 7—figure supplement 4.**
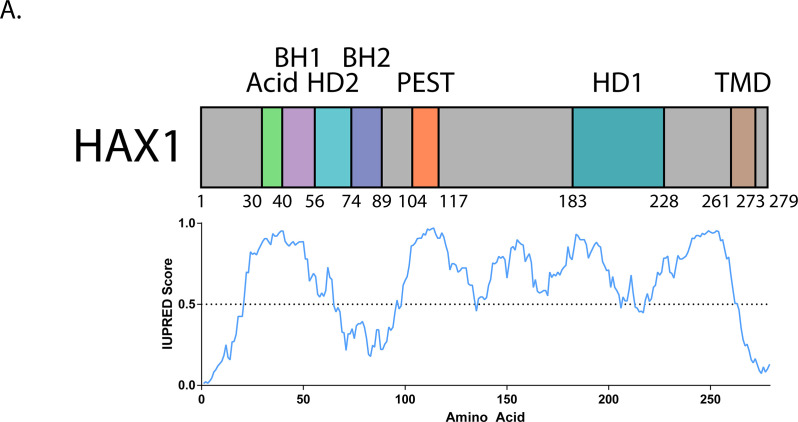
HAX1 is a highly disordered protein. (**A**) Domain map of HAX1 with IUPRED disorder prediction score plotted underneath (Mészáros et al., 2018; Mészáros et al., 2009). IUPRED scores higher than 0.5 predict disorder. Analysis suggests that HAX1 is a highly disordered protein. Acidic domain labeled in green, BH1 and BH2 domains labeled in purple, HD1 and HD2 domains labeled in blue, PEST domain labeled in orange, and transmembrane domain (TMD) labeled in tan.

Specifically, we find that HAX1, OPA1, PHB2, PARL, SMAC/DIABLO, and HTRA2 are more insoluble, which implicates a key role for Skd3 in regulating apoptotic and proteolytic pathways (Baumann et al., 2014; Chai et al., 2000; Chao et al., 2008; Cipolat et al., 2006; Frezza et al., 2006; Klein et al., 2007; Saita et al., 2017; Yoshinaka et al., 2019). Additionally, the regulators of the mitochondrial calcium uniporter (MCU), MICU1 and MICU2 along with several members of the SLC25 family (including the calcium binding SLC25A25 and SLC25A13) were found to be more insoluble in the knockout compared to the wild type, implicating Skd3 in the regulation of mitochondrial calcium transport and signaling (Anunciado-Koza et al., 2011; Csordás et al., 2013; Palmieri et al., 2001; Patron et al., 2014; Perocchi et al., 2010; Plovanich et al., 2013). Indeed, a sedimentation assay as described in Figure 7a in combination with western blot analysis shows an overall decrease in solubility of MICU2 in mitochondria lacking Skd3 (Figure 7—figure supplement 3a,b). We also observed an enrichment of translocase of the inner membrane (TIM) components, TIMM8A, TIMM8B, TIMM13, TIMM21, TIMM22, TIMM23, and TIMM50 in the insoluble fraction of Skd3 knockouts (Chacinska et al., 2005; Donzeau et al., 2000; Geissler et al., 2002; Meinecke et al., 2006; Mokranjac et al., 2003; Paschen et al., 2000; Sirrenberg et al., 1996; Yamamoto et al., 2002). Finally, we observed an enrichment in respiratory complex I and III proteins and their assembly factors such as NDUFA8, NDUFA11, NDUFA13, NDUFB7, NDUFB10, TTC19, COX11, and CYC1 in the insoluble fraction of Skd3 knockouts (Figure 6b and Supplementary file 2; Angebault et al., 2015; Ghezzi et al., 2011; Spinazzi et al., 2019; Szklarczyk et al., 2011; Tzagoloff et al., 1990). Overall, these results suggest an important role for Skd3 in maintaining the solubility of proteins of the inner mitochondrial membrane and intermembrane space, including key regulators in apoptosis, mitochondrial calcium regulation, protein import, and respiration. Thus, in cells Skd3 appears critical for protein solubility in the intermembrane space and mitochondrial inner membrane.

### Skd3 promotes HAX1 solubility in human cells

HAX1 is a highly-disordered protein that has been previously identified as a Skd3 substrate both in cells and in silico (Figure 7—figure supplement 4; Chen et al., 2019; Wortmann et al., 2015). HAX1 is an anti-apoptotic BCL-2 family protein that enables efficient cleavage of HTRA2 by PARL to promote cell survival (Chao et al., 2008; Klein et al., 2007). To test if Skd3 regulates HAX1 solubility in human cells, we compared the solubility of HAX1 in wild-type and Skd3-knockout HAP1 cells via sedimentation analysis and western blot. In wild-type cells, HAX1 remained predominantly soluble (Figure 7d,e). However, when Skd3 was deleted HAX1 became predominantly insoluble (Figure 7d,e). Thus, Skd3 is essential for HAX1 solubility in cells. Curiously, loss of Skd3 has been previously shown to promote apoptosis in specific contexts (Chen et al., 2019). Furthermore, HAX1 stability has been implicated as a regulator of apoptotic signaling (Baumann et al., 2014). Our data support a model whereby Skd3 exerts its anti-apoptotic effect by maintaining HAX1 solubility and contingent functionality.

### MGCA7-linked Skd3 variants display diminished disaggregase activity

**Figure 8.**
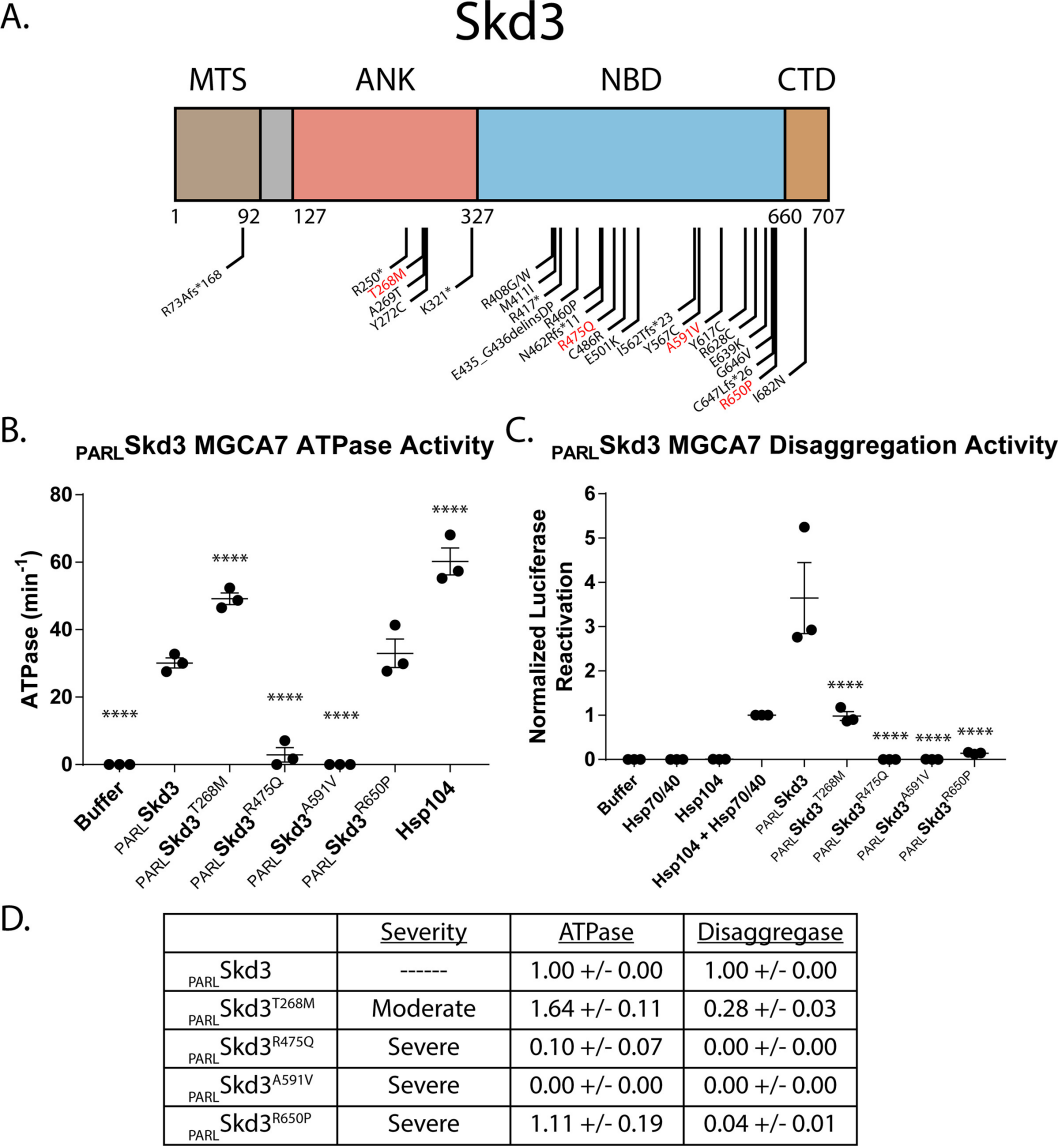
Skd3 disaggregase activity predicts the clinical severity of MGCA7-associated mutations. (**A**) Domain map depicting all published mutations in Skd3 that have been associated with MGCA7. Mutants in red are studied further here. (**B**) ATPase assay showing the effect of four homozygous MGCA7 mutations on Skd3 activity. _PARL_Skd3^T268M^ has increased ATPase activity, _PARL_Skd3^R475Q^ and _PARL_Skd3^A591V^ have decreased ATPase activity, and _PARL_Skd3^R650P^ has unchanged ATPase activity compared to wild type. _PARL_Skd3 MGCA7 mutants ATPase activities were compared to _PARL_Skd3 wild-type using one-way ANOVA and a Dunnett’s multiple comparisons test (N = 3, individual data points shown as dots, bars show mean ± SEM, ****p<0.0001). (**C**) Luciferase disaggregation/reactivation assay showing the effect of the same four homozygous MGCA7 mutations on Skd3 activity. _PARL_Skd3^T268M^ had reduced disaggregase activity, whereas _PARL_Skd3^R475Q^, _PARL_Skd3^A591V^, and _PARL_Skd3^R650P^were almost completely inactive compared to wild type. Luciferase activity was buffer subtracted and normalized to Hsp104 plus Hsp70 and Hsp40. Luciferase disaggregase activity was compared to _PARL_Skd3 wild type using one-way ANOVA and a Dunnett’s multiple comparisons test (N = 3, individual data points shown as dots, bars show mean ± SEM, ****p<0.0001). (**D**) Table summarizing the clinical severity of each MGCA7 mutation as well as the ATPase activity and luciferase disaggregase activity. The table shows a relationship between luciferase disaggregase activity and clinical severity, but no relationship between either the ATPase activity and clinical severity or ATPase and luciferase disaggregase activity. Values represent ATPase activity and luciferase disaggregase activity normalized to wild-type _PARL_Skd3 activity. Values show mean ± SEM (N = 3).

Skd3 has been implicated in a severe mitochondrial disorder, MGCA7, yet little is known about its contribution or function in this disease (Capo-Chichi et al., 2015; Kanabus et al., 2015; Kiykim et al., 2016; Pronicka et al., 2017; Saunders et al., 2015; Wortmann et al., 2016; Wortmann et al., 2015). Indeed, many mutations in Skd3 are connected with MGCA7 (Figure 8a). Most of these are clustered in the NBD, but several are also in the ankyrin-repeat domain, and one frameshift is found in the mitochondrial targeting signal (Figure 8a). Some MGCA7-linked Skd3 variants, such as R250* and K321* (where * indicates a stop codon), lack the NBD and would be predicted in light of our findings to be incapable protein disaggregation and indeed any ATPase-dependent activities (Figure 5b,c). We hypothesized that MGCA7-linked missense mutations also directly affect Skd3 disaggregase activity. To test this hypothesis, we purified MGCA7-linked variants of Skd3 from cases where both patient alleles bear the mutation, specifically: T268M, R475Q, A591V, and R650P (Pronicka et al., 2017). These Skd3 variants cause MGCA7 on a spectrum of clinical severity, which is defined by a scoring system assigned by physicians based on the presentation of various symptoms associated with MGCA7 (Pronicka et al., 2017). Broadly, the severe phenotype often presented with the absence of voluntary movements in neonates, hyperexcitability, ventilator dependency, and early death (Pronicka et al., 2017). The ankyrin-repeat variant, T268M, is linked to moderate MGCA7, whereas the NBD variants (R475Q, A591V, and R650P) are linked to severe MGCA7 (Pronicka et al., 2017).

Surprisingly, the ATPase activity varied for each MGCA7-linked variant. T268M had significantly increased ATPase activity, R475Q and A591V had marked decreased ATPase activity, and R650P ATPase was indistinguishable from wild type (Figure 8b). These ATPase activities did not correlate with clinical severity (Figure 8d; Pronicka et al., 2017). Thus, Skd3 variant ATPase activity does not accurately predict MGCA7 severity, as the mutation associated with mild MGCA7 had elevated ATPase relative to wild type, whereas different mutations capable of causing severe MGCA7 could exhibit impaired or nearly wild-type ATPase activity.

To address the disconnect between ATPase activity and MGCA7 disease severity, we next tested the disaggregase activity of these MGCA7-linked variants. Strikingly, and in contrast to ATPase activity, we found disaggregase activity tracks closely with disease severity. T268M, the only moderate phenotype variant tested, had ~27% disaggregase activity compared to wild type. By contrast, the three severe MGCA7 variants, R475Q, A591V, and R650P abolish or diminish disaggregation activity with 0%, 0%, and ~4% disaggregation activity compared to wild type, respectively (Figure 8c). Thus, disaggregase activity but not ATPase activity, is tightly correlated with clinical severity of MGCA7-linked mutations (Figure 8d; Pronicka et al., 2017). It will be of interest to test further MGCA7-linked variants to determine whether this trend holds. Nevertheless, our findings suggest that defects in Skd3 protein-disaggregase activity (and not other ATPase-related functions) are the driving factor in MGCA7 and pivotal to human health.

## Discussion

**Figure 9.**
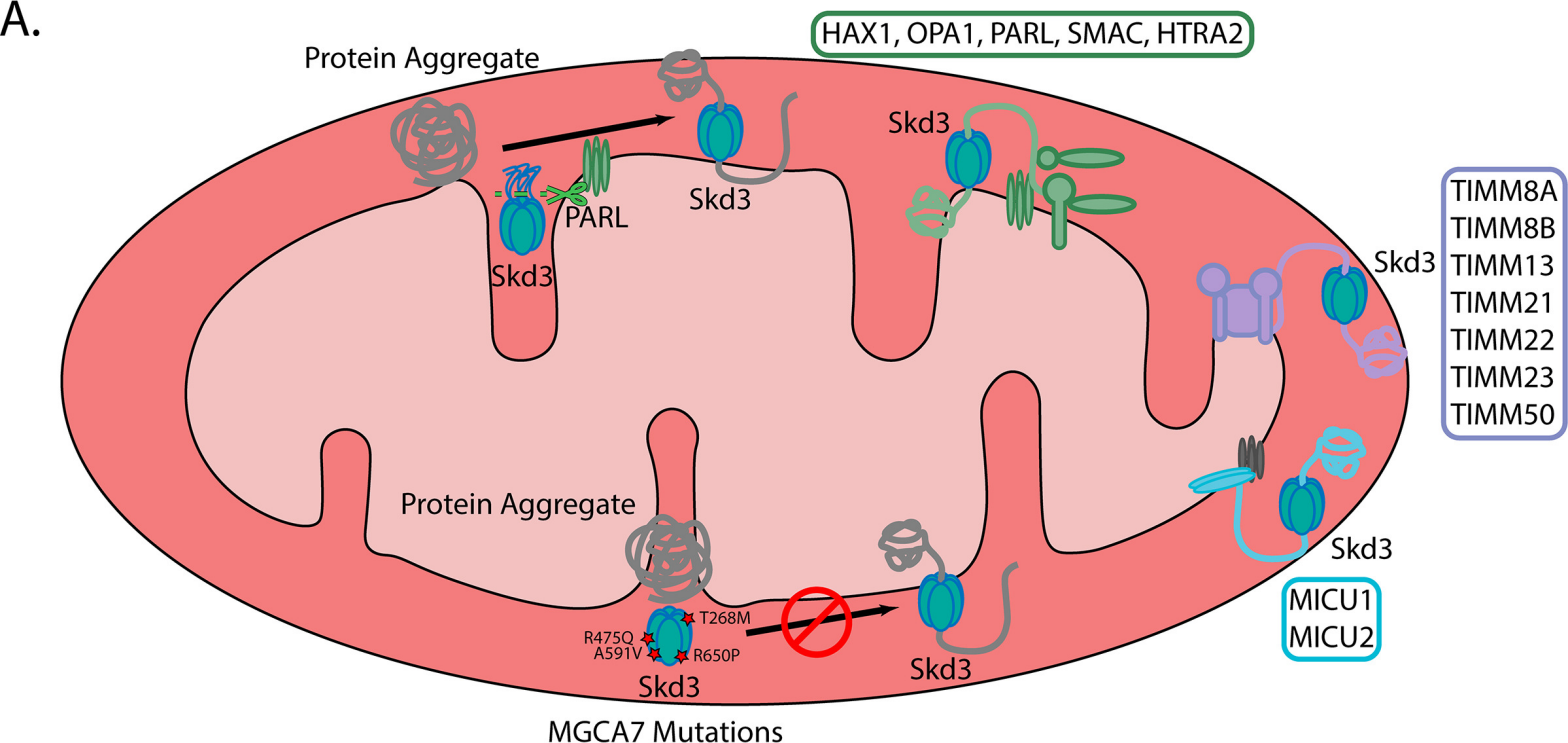
Skd3 is a protein disaggregase that is activated by PARL and inactivated by MGCA7-linked mutations. (**A**) Schematic illustrating (**i**) that Skd3 is a protein disaggregase that is activated by PARL cleavage of a hydrophobic auto-inhibitory peptide, (ii) that Skd3 works to solubilize key substrates in the mitochondrial intermembrane space and inner membrane that are involved in apoptosis, protein import, calcium handling, and respiration, and (iii) that mutations in Skd3 associated with MGCA7 result in defective Skd3 disaggregase activity in a manner that predicts the clinical severity of disease.

At the evolutionary transition from protozoa to metazoa, the potent mitochondrial AAA+ protein disaggregase, Hsp78, was lost (Erives and Fassler, 2015). Thus, it has long remained unknown whether metazoan mitochondria disaggregate and reactivate aggregated proteins. Here, we establish that another AAA+ protein, Skd3, is a potent metazoan mitochondrial protein disaggregase. Skd3 is activated by a mitochondrial inner-membrane rhomboid protease, PARL (Figure 9). PARL removes a hydrophobic auto-inhibitory sequence from the N-terminal region of Skd3, which prior to cleavage may limit Skd3 interactions with substrate (Figure 9). In this way, Skd3 only becomes fully activated as a disaggregase once it reaches its final cellular destination. Thus, cells might avoid any potential problems that could arise from unchecked Skd3 activity in the wrong cellular location. Skd3 activation by PARL may underlie several potential physiological mechanisms whereby Skd3 is either activated by PARL in response to certain cellular stressors or Skd3 is no longer activated by PARL upon the onset of apoptotic signaling.

Skd3 couples ATP binding and hydrolysis to protein disaggregation. To do so, Skd3 utilizes conserved AAA+ motifs in its NBD, including the Walker A and B motifs to bind and hydrolyze ATP, as well as a conserved pore-loop tyrosine, which likely engages substrate directly in a manner similar to other HCLR clade AAA+ proteins (Erzberger and Berger, 2006; Gates et al., 2017; Lopez et al., 2020; Martin et al., 2008; Rizo et al., 2019; Seraphim and Houry, 2020). However, the isolated NBD is insufficient for disaggregase activity, which indicates an important role for the ankyrin-repeat domain. Intriguingly, an ankyrin-repeat domain is also important for the activity of an unrelated ATP-independent protein disaggregase, cpSRP43, where it makes critical contacts with substrate (Jaru-Ampornpan et al., 2013; Jaru-Ampornpan et al., 2010; McAvoy et al., 2018; Nguyen et al., 2013). Thus, ankyrin-repeat domains appear to be a recurring feature of diverse protein disaggregases.

Importantly, Skd3 is a stand-alone disaggregase and does not require Hsp70 and Hsp40 for maximal activity. This finding is consistent with the absence of Hsp70-interacting domains (NTD, NBD1, and MD) found in Hsp104, which enable collaboration with Hsp70 (DeSantis et al., 2014; Lee et al., 2013; Sweeny et al., 2015; Sweeny et al., 2020). Future structural and biochemical studies will further inform our mechanistic understanding of Skd3 disaggregase activity.

We establish that Skd3 can disaggregate disease-causing α-synuclein fibrils in vitro, demonstrating its robust activity as a disaggregase and identifying it as a potential therapeutic for synucleinopathies. The realization that human cells harbor a AAA+ protein disaggregase of greater potency than Hsp104 opens several therapeutic opportunities. Indeed, Skd3 is expressed in neurons and shifting localization of activated Skd3 to the cytoplasm could help combat cytoplasmic aggregates. Likewise, the expression of the _PARL_Skd3 enhanced variant in the cytoplasm of dopaminergic neurons may elicit therapeutic benefit similar to Hsp104 and engineered variants in Parkinson’s disease models (Jackrel et al., 2014; Lo Bianco et al., 2008; Mack et al., 2020; March et al., 2020; Tariq et al., 2019). Future studies will further inform our understanding of how to harness Skd3 disaggregase activity therapeutically in synucleinopathies such as Parkinson’s disease and other neurodegenerative diseases connected with aberrant protein aggregation.

We demonstrate that Skd3 is essential for maintaining the solubility of mitochondrial inner-membrane and intermembrane space protein complexes and specifically maintains solubility of the anti-apoptotic protein HAX1 in human cells (Figure 9). We suggest that HAX1 solubility is important for its anti-apoptotic effect. The precise mechanism of regulation between Skd3 and HAX1, PARL, OPA1, HTRA2, and SMAC/DIABLO warrants future study (Figure 9).

In addition to finding that many human mitochondrial proteins are more insoluble in the absence of Skd3, a small fraction of proteins are more insoluble in the presence of Skd3. Closer analysis of these enriched proteins suggests that many are mitochondrial matrix-associated, especially mitoribosome proteins (Figure 7b, Supplementary file 1). The mitoribosome is a large, megadalton sized protein complex that is much more proteinaceous than its cytoplasmic counterpart and assembles into larger polysomes during active translation (Couvillion et al., 2016; Greber and Ban, 2016; Saurer et al., 2019). Thus, changes in solubility of the mitoribosome components could be due to increased mitoribosome or polysome assembly in the presence of Skd3.

It is surprising that Skd3 maintains protein solubility in the mitochondrial intermembrane space and inner membrane, as Hsp78 is found in the mitochondrial matrix (Bateman et al., 2002; Germaniuk et al., 2002; Moczko et al., 1995; Rottgers et al., 2002; Schmitt et al., 1995; von Janowsky et al., 2006). Since Skd3 appears in evolution alongside Hsp78 in choanoflagellates it may have initially arisen to serve a distinct function. We hypothesize that the increasing number and complexity of mitochondrial inner membrane protein assemblies (such as MICU1/MICU2/MCU and respiratory complex I) in metazoa might necessitate the requirement of Skd3 activity in the inner mitochondrial membrane and intermembrane space to maintain proteostasis in these compartments (Botham et al., 2019; Hung et al., 2014; Yoshinaka et al., 2019).

Hsp78 is known to be involved in mitochondrial protein import in yeast (Moczko et al., 1995; Schmitt et al., 1995). Furthermore, the ankyrin-repeat containing chloroplast disaggregase, cpSRP43, is involved in sorting membrane-targeted cargo (Jaru-Ampornpan et al., 2013; Jaru-Ampornpan et al., 2010). It is unknown if Skd3 participates in protein import and sorting in mitochondria. However, given the involvement of two closely related organelle-specific disaggregases in protein import and sorting and the changes in solubility of key TIM components in the absence of Skd3, it is plausible that Skd3 might play a role in protein import and sorting in mitochondria (Figure 7b,c). Mitochondrial presequences are prone to aggregation and the accumulation of uncleaved precursor aggregates can lead to the activation of an early mitochondrial unfolded protein response (mtUPR) (Endo et al., 1995; Poveda-Huertes et al., 2020; Yano et al., 2003). Skd3 might be required to keep aggregation-prone presequence signals in an unfolded state during import and sorting prior to cleavage or may help disaggregate them if they aggregate. Further investigation is required to determine which changes in protein solubility are driven by defects in Skd3 activity and which are from potential defects in mitochondrial protein import or membrane insertion.

Mutations in Skd3 are connected to MGCA7, which can be a devastating disorder involving severe neurologic deterioration, neutropenia, and death in infants (Wortmann et al., 2016). Importantly, we establish that diverse MGCA7-linked mutations in Skd3 impair disaggregase activity, but not necessarily ATPase activity (Figure 9). The degree of impaired disaggregase activity predicts the clinical severity of the disease, which suggests that disaggregase activity is a critical factor in disease. However, it is yet unclear which Skd3 substrate or substrates contribute to the MGCA7 etiology. Our mass-spectrometry data suggest that MGCA7 arises due to severely compromised proteostasis in the mitochondrial inner-membrane and intermembrane space (Figure 9). Hence, small-molecule drugs that restore wild-type levels of disaggregase activity to MGCA7-linked Skd3 variants could be valuable therapeutics.

Finally, Skd3 has also emerged as a factor in Venetoclax resistance, a FDA-approved drug for the treatment of acute myeloid leukemia (AML), which exerts its mechanism via BCL-2 inhibition (Chen et al., 2019). These studies suggest that inhibition of Skd3 may be of critical therapeutic importance for treating Venetoclax-resistant cancers (Chen et al., 2019). Small-molecule screens targeted at finding inhibitors of Skd3 disaggregase activity may yield important drugs for Venetoclax-resistant AML patients. Thus, the Skd3 disaggregase assays established in this study could provide a powerful platform for isolating therapeutic compounds for MGCA7 and AML.

## Materials and methods

### Multiple sequence alignments

NBD sequences were acquired via UniProtKB for *Homo sapiens* Skd3, *Saccharomyces cerevisiae* Hsp104, *Saccharomyces cerevisiae* Hsp78, *Escherichia coli* ClpB, *Escherichia coli* ClpA, and *Staphylococcus aureus* ClpC. Skd3 sequences were acquired via SMART protein domain annotation resource (Letunic and Bork, 2018). Sequences from *Anolis carolinensis*, *Bos taurus*, *Callithrix jacchus*, *Canis lupus*, *Capra hircus*, *Danio rerio*, *Equus caballus*, *Geospiza fortis*, *Gorilla gorilla*, *Homo sapiens*, *Monosiga brevicollis*, *Mus musculus*, *Nothobranchius rachovii*, *Rattus norvegicus*, *Sus scrofa*, *Trachymyrmex septentrionalis*, *Trichinella papuae*, and *Xenopus laevis* were used to generate alignment for Figure 1 and Supplementary file 1. Compiled sequences were aligned and made into a guide tree via Clustal Omega (Madeira et al., 2019). Alignment image was generated via BoxShade tool (Hofmann and Baron, 1996). Guide tree image was built using FigTree (Rambaut, 2012). Species images were used under license via PhyloPic. Sequence logo was created using WebLogo and 42 mammalian Skd3 protein sequences (*Ailuropoda melanoleuca*, *Callorhinus ursinus*, *Canis lupus*, *Carlito syrichta*, *Cebus capucinus*, *Ceratotherium simum*, *Cercocebus atys*, *Chlorocebus sabaeus*, *Colobus angolensis*, *Equus asinus, Equus caballus*, *Equus przewalskii*, *Felis catus*, *Gorilla gorilla*, *Gulo gulo*, *Grammomys surdaster*, *Homo sapiens*, *Macaca fascicularis*, *Macaca mulatta*, *Macaca nemestrina*, *Mandrillus leucophaeus*, *Microcebus murinus*, *Microtus ochrogaster*, *Mustela putorius*, *Nomascus leucogenys*, *Odobenus rosmarus*, *Orycteropus afer*, *Pan paniscus*, *Pan troglodyte*, *Panthera tigris*, *Papio anubis*, *Piliocolobus tephrosceles*, *Pongo abelii*, *Propithecus coquereli*, *Puma concolor*, *Rhinopithecus bieti*, *Rhinopithecus roxellana*, *Rousettus aegyptiacus*, *Theropithecus gelada*, *Ursus arctos*, *Ursus maritimus*, and *Zalophus californianus*) (Crooks et al., 2004; Schneider and Stephens, 1990).

### Cloning MBP-Skd3 plasmids

_MPP_Skd3, _PARL_Skd3, _ANK_Skd3, and _NBD2_Skd3 were cloned into the pMAL C2 plasmid with TEV site (Yoshizawa et al., 2018) using Gibson assembly (Gibson et al., 2009). The mitochondrial targeting signal was identified using MitoProt in agreement with previous work (Claros and Vincens, 1996; Wortmann et al., 2015). Site-directed mutagenesis was performed using QuikChange site-directed mutagenesis (Agilent) and confirmed by DNA sequencing.

### Purification of Skd3

Skd3 variants were expressed as an N-terminally MBP-tagged protein in BL21 (DE3) RIL cells (Agilent). Cells were lysed via sonication in 40 mM HEPES-KOH pH = 7.4, 500 mM KCl, 20% (w/v) glycerol, 5 mM ATP, 10 mM MgCl_2_, 2 mM β-mercaptoethanol, 2.5 µM PepstatinA, and cOmplete Protease Inhibitor Cocktail (one tablet/250 mL, Millipore Sigma). Lysates were centrifuged at 30,597xg and 4°C for 20 min and the supernatant was applied to amylose resin (NEB). The column was washed with 15 column volumes (CV) of wash buffer (WB: 40 mM HEPES-KOH pH = 7.4, 500 mM KCl, 20% (w/v) glycerol, 5 mM ATP, 10 mM MgCl_2_, 2 mM β-mercaptoethanol, 2.5 µM PepstatinA, and cOmplete Protease Inhibitor Cocktail) at 4°C, 3 CV of WB supplemented with 20 mM ATP at 25°C for 30 min, and 15 CV of WB at 4°C. The protein was then exchanged into elution buffer (EB: 50 mM Tris-HCl pH = 8.0, 300 mM KCl, 10% glycerol, 5 mM ATP, 10 mM MgCl_2_, and 2 mM β-mercaptoethanol) with 8 CV and eluted via TEV cleavage at 34°C. The protein was then run over a size exclusion column (GE Healthcare HiPrep 26/60 Sephacryl S-300 HR) in sizing buffer (50 mM Tris-HCl pH = 8.0, 500 mM KCl, 10% glycerol, 1 mM ATP, 10 mM MgCl_2_, and 1 mM DTT). Peak fractions were collected, concentrated to >5 mg/mL, supplemented with 5 mM ATP, and snap frozen. Protein purity was determined to be >95% by SDS-PAGE and Coomassie staining.

### Purification of Hsp104

Hsp104 was purified as previously described (DeSantis et al., 2014). In brief, Hsp104 was expressed in BL21 (DE3) RIL cells, lysed via sonication in lysis buffer [50 mM Tris-HCl pH = 8.0, 10 mM MgCl_2_, 2.5% glycerol, 2 mM β-mercaptoethanol, 2.5 µM PepstatinA, and cOmplete Protease Inhibitor Cocktail (one mini EDTA-free tablet/50 mL, Millipore Sigma)], clarified via centrifugation at 30,597xg and 4°C for 20 min, and purified on Affi-Gel Blue Gel (Bio-Rad). Hsp104 was eluted in elution buffer (50 mM Tris-HCl pH = 8.0, 1M KCl, 10 mM MgCl_2_, 2.5% glycerol, and 2 mM β-mercaptoethanol) and then exchanged into storage buffer (40 mM HEPES-KOH pH = 7.4, 500 mM KCl, 20 mM MgCl_2_, 10% glycerol, 1 mM DTT). The protein was diluted to 10% in buffer Q (20 mM Tris-HCl pH = 8.0, 50 mM NaCl, 5 mM MgCl_2_, and 0.5 mM EDTA) and loaded onto a 5 mL RESOURCE Q anion exchange chromatography (GE Healthcare). Hsp104 was eluted via linear gradient of buffer Q+ (20 mM Tris pH = 8.0, 1M NaCl, 5 mM MgCl_2_, and 0.5 mM EDTA). The protein was then exchanged into storage buffer and snap frozen. Protein purity was determined to be >95% by SDS-PAGE and Coomassie staining.

### Purification of Hsc70 and Hdj1

Hsc70 and Hdj1 were purified as previously described (Michalska et al., 2019). Hsc70 and Hdj1 were expressed in BL21 (DE3) RIL cells with an N-terminal His-SUMO tag. Cells were lysed via sonication into lysis buffer [50 mM HEPES-KOH pH = 7.5, 750 mM KCl, 5 mM MgCl_2_, 10% glycerol, 20 mM imidazole, 2 mM β-mercaptoethanol, 5 µM pepstatin A, and cOmplete Protease Inhibitor Cocktail (one mini EDTA-free tablet/50 mL)]. Lysates were centrifuged at 30,597xg and 4°C for 20 min and the supernatant was bound to Ni-NTA Agarose resin (Qiagen), washed with 10 CV of wash buffer (50 mM HEPES-KOH pH = 7.5, 750 mM KCl, 10 mM MgCl_2_, 10% glycerol, 20 mM imidazole, 1 mM ATP, and 2 mM β-mercaptoethanol), and then eluted with 2 CV of elution buffer (wash buffer supplemented with 300 mM imidazole). The tag was removed via Ulp1 (1:100 Ulp1:Protein molar ratio) cleavage during dialysis into wash buffer. The protein was further purified via loading onto a 5 mL HisTrap HP column (GE Healthcare) and pooling the untagged elution. Cleaved protein was pooled, concentrated, purified further by Resource Q ion exchange chromatography, and snap frozen. Protein purity was determined to be >95% by SDS-PAGE and Coomassie staining.

### ATPase assays

Proteins (0.25 µM monomer) were incubated with ATP (1 mM) (Innova Biosciences) at 37°C for 5 min (or otherwise indicated) in luciferase reactivation buffer (LRB; 25 mM HEPES-KOH [pH = 8.0], 150 mM KAOc, 10 mM MgAOc, 10 mM DTT). For substrate-stimulation of ATPase activity the indicated concentration of substrate was added. ATPase activity was assessed via inorganic phosphate release with a malachite green detection assay (Expedeon) and measured in Nunc 96 Well Optical plates on a Tecan Infinite M1000 plate reader. Background hydrolysis was measured at time zero and subtracted (DeSantis et al., 2012).

### Luciferase disaggregation and reactivation assays

Firefly luciferase aggregates were formed by incubating luciferase (50 µM) in LRB (pH=7.4) plus 8M urea at 30°C for 30 min. The luciferase was then rapidly diluted 100-fold into LRB, snap frozen, and stored at −80°C until use. Hsp104 and Skd3 variants (1 µM monomer, unless otherwise indicated) were incubated with 50 nM aggregated firefly luciferase in the presence or absence of Hsc70 and Hdj2 (0.167 µM each) in LRB plus 5 mM ATP plus an ATP regeneration system (ARS; 1 mM creatine phosphate and 0.25 µM creatine kinase) at 37°C for 90 min (unless otherwise indicated). The nucleotide-dependence of Skd3 disaggregation activity was tested in the presence of ATP (Sigma), AMP-PNP (Roche), ATPγS (Roche), or ADP (MP Biomedicals) for 30 min at 37°C without ARS. Recovered luminescence was monitored in Nunc 96 Well Optical plates using a Tecan Infinite M1000 plate reader (DeSantis et al., 2012).

### α-Synuclein disaggregation assay

α-Synuclein fibrils were acquired from the Luk lab and formed as previously described (Luk et al., 2012). _PARL_Skd3 (10 μM monomer) was incubated with α-synuclein fibrils (0.5 μM monomer) in LRB in the presence or absence of ARS (10 mM ATP, 10 mM creatine phosphate, 40 μg/mL creatine kinase) at 37°C for 90 min. The samples were then centrifuged at 4°C and 20,000xg for 20 min. After centrifugation the supernatants were pipetted off of the pellets and the pellets were boiled in Pellet Buffer (PB; 50 mM Tris-HCl [pH = 8.0], 8M Urea, 150 mM NaCl, 10 uL/1 mL mammalian PI cocktail [Sigma CAT# P8340]) for 5 min at 99°C. The total sample, supernatant, and pellet samples were then blotted on nitrocellulose membrane (ThermoFisher Scientific CAT# 88018) and incubated with the SYN211 antibody (ThermoFisher Scientific CAT# AHB0261). Blots were then incubated with the IRDye 800CW Goat anti-Mouse IgG Secondary Antibody (Li-COR CAT# 926–32210) and imaged using the Li-Cor Odyssey Fc Imaging System. Samples were quantified using FIJI and normalized as (signal in supernatant)/(signal in pellet + signal in supernatant).

### Western blots

Mammalian whole cell lysates were prepared by boiling 500,000 cells in 1x Sample Buffer (SB; 60 mM Tris-HCl [pH = 6.8], 5% glycerol, 2% SDS, 10% β-mercaptoethanol, 0.025% bromophenol blue, 1x Mammalian PI cocktail) for 5 min at 99°C. Sedimentation assay samples were prepared as described above. Western blot samples were boiled for 5 min at 99°C in 1x SB, separated by SDS-PAGE on a gradient gel (4%–20%, Bio-Rad CAT# 3450033), and transferred to a PVDF membrane. Membranes were blocked in Odyssey Blocking Buffer in PBS (Li-Cor CAT# 927–40000) for 1 hr at 25°C. Blots were then incubated in primary antibody overnight at 4°C and then in secondary for 30 min at 25°C. The antibodies used were: α-CLPB (Abcam CAT# ab235349), α-HAX1 (Abcam CAT# ab137613), α-COXIV (Abcam CAT# ab14744), α-GAPDH (Abcam CAT# ab8245), α-MICU2 (Abcam CAT# ab101465), IRDye 800CW Goat α-mouse secondary (Li-Cor CAT# 926–32210), and IRDye 680RD Goat α-rabbit secondary (Li-Cor CAT# 926–68071). Blots were imaged on a Li-Cor Odyssey Fc Imaging System.

### Mammalian cell culture

Isogenic HAP1 and HAP1 Δ*CLPB* cells were acquired directly from Horizon Discovery (CAT# HZGHC81570 and HZGHC007326c001) and have been control quality checked by the vendor. HAP1 cells are a human near-haploid cell line derived from the male chronic myelogenous leukemia (CML) cell line KBM-7 (Essletzbichler et al., 2014). In brief, the *CLPB* knockout line was generated via CRISPR-Cas9 and the gRNA TGGCACGGGCAGCTTCCAAC to make a 1 bp insertion into exon 2 of the *CLPB* gene. Knockout was confirmed by Sanger sequencing (DNA) by the vendor and western blot (Figure 7—figure supplement 1). Cells were maintained in IMDM (Gibco CAT# 12440053) supplemented with 10% FBS (GE CAT# SH3007003) and 1% P/S (Gibco CAT# 15140122) at 37°C and 5% CO_2_. Cells were grown at a confluency of 50–60% for mitochondrial isolation.

### Mitochondrial isolation

Mitochondria were isolated as previously described (Frezza et al., 2007). In brief, 50–100*10^6^ cells were resuspended in 5 mL SM buffer (50 mM Tris-HCl [pH = 7.4], 0.25M sucrose, 2 mM EDTA, and 1% BSA) and homogenized with a Dounce homogenizer and Teflon pestle (30 strokes at 600 RPM) at 4°C. Lysate was then centrifuged at 600xg for 10 min. The supernatant was collected, and the pellet was dissolved in 5 mL SM buffer and homogenized (15 strokes at 600 RPM). Sample was then centrifuged at 600xg for 10 min and the supernatant was pooled and centrifuged at 12,000xg for 15 min. The pellet was collected and used for further experiments.

### Mitochondrial sedimentation assay

Mitochondrial sedimentation assay was performed essentially as previously described (Wilkening et al., 2018). 60–80 μg isolated mitochondria were resuspended in 200 μL Mitochondrial Resuspension Buffer (40 mM HEPES-KOH, pH = 7.6, 500 mM sucrose, 120 mM K-acetate, 10 mM Mg-acetate, 5 mM glutamate, 5 mM malate, 5 mM EDTA, 5 mM ATP, 20 mM creatine phosphate, 4 μg/mL creatine kinase, 1 mM DTT) and incubated at 37°C for 20 min. The mitochondria were then pelleted at 12,000xg for 10 min at 4°C. The mitochondria were then resuspended in 200 μL Lysis Buffer (30 mM Tris-HCl, pH = 7.4, 200 mM KCl, 0.5% v/v Triton X-100, 5 mM EDTA, 0.5 mM PMSF, 1x Mammalian PI cocktail) and lysed in a thermomixer at 2,000 RPM for 10 min at 4°C. The protein concentration of the lysate was then quantified using a BCA assay (ThermoFisher CAT# 23225). 12 μg of lysate was added to a total volume of 50 μL Lysis Buffer and reserved as a total protein sample. 12 μg of lysate was added to a total volume of 50 μL Lysis Buffer and sedimented at 20,000xg for 20 min at 4°C. The supernatant was removed, TCA precipitated, and frozen for later processing. The pellet was boiled in 10 μL of Pellet Buffer and frozen for later processing.

### Mass spectrometry

Pellet samples were excised as whole lanes from gels, reduced with TCEP, alkylated with iodoacetamide, and digested with trypsin. Tryptic digests were desalted by loading onto a MonoCap C18 Trap Column (GL Sciences), flushed for 5 min at 6 μL/min using 100% Buffer A (H_2_0, 0.1% formic acid), then analyzed via LC (Waters NanoAcquity) gradient using Buffer A and Buffer B (acetonitrile, 0.1% formic acid) (initial 5% B; 75 min 30% B; 80 min 80% B; 90.5–105 min 5% B) on the Thermo Q Exactive HF mass spectrometer. Data were acquired in data-dependent mode. Analysis was performed with the following settings: MS1 60K resolution, AGC target 3e6, max inject time 50 ms; MS2 Top N = 20 15K resolution, AGC target 5e4, max inject time 50 ms, isolation window = 1.5 m/z, normalized collision energy 28%. LC-MS/MS data were searched with full tryptic specificity against the UniProt human database using MaxQuant 1.6.8.0. MS data were also searched for common protein N-terminal acetylation and methionine oxidation. Protein and peptide false discovery rate was set at 1%. LFQ intensity was calculated using the MaxLFQ algorithm (Cox et al., 2014). Fold enrichment was calculated as LFQ intensity from the Δ*CLPB* pellet divided by the LFQ intensity from the wild-type pellet. High confidence hits were quantified as minimum absolute fold change of 2 and p-value<0.05.

## Data Availability

All data generated or analysed during this study are included in the manuscript and supporting files.

## Additional files

Supplementary file 1. Alignment of Skd3 to diverse metazoan lineages shows conservation of key motifs and domains. Alignment of Skd3 protein from diverse metazoan lineages. Alignment was constructed using Clustal Omega. Alignment shows high level of conservation of Skd3 among species. *H. sapiens*, *G. gorilla*, and *C. jacchus* Skd3 have an additional insertion in the ankyrin-repeat domain that is not conserved in the other species. This alignment was used to generate the phylogenetic tree in Figure 1B. The protozoan *M. brevicollis* Skd3 sequence was included in the alignment for reference. MTS (mitochondrial-targeting sequence, ANK (ankyrin-repeat domain), NBD (nucleotide-binding domain), and CTD (C-terminal domain).

Supplementary file 2. Proteins enriched in HAP1 *ΔCLPB* pellet. Proteins from mass spectrometry data in Figure 7b highlighted in red that have >2.0 fold change increase in the *ΔCLPB* insoluble fraction compared to wild type and a p-value of <0.05.

Supplementary file 3. Proteins enriched in HAP1 WT pellet. Proteins from mass spectrometry data in Figure 7b highlighted in green that have >2.0 fold change increase in the wild-type insoluble fraction compared to *ΔCLPB* and a p-value of <0.05.

Transparent reporting form

## Appendix 1

**Appendix 1—key resources table:**

Reagent type (species) or resource	Designation	Source or reference	Identifiers	Additional information
Antibody	Mouse monoclonal α-α-synuclein (SYN211)	ThermoFisher Scientific	AHB0261; RRID:AB_10978319	(1:500 dilution)
Antibody	Rabbit polyclonal α-CLPB (Skd3)	abcam	ab235349; RRID:AB_2847899	(1:1000 dilution)
Antibody	Rabbit polyclonal α-CLPB (Skd3)	abcam	ab76179; RRID:AB_1310087	(1:1000 dilution)
Antibody	Rabbit polyclonal α-CLPB (Skd3)	abcam	ab87253; RRID:AB_1952530	(1:1000 dilution)
Antibody	Rabbit polyclonal α-CLPB (Skd3)	Proteintech	15743–1-AP; RRID:AB_2847900	(1:1000 dilution)
Antibody	Mouse monoclonal α-COXIV	abcam	ab14744; RRID:AB_301443	(1:1000 dilution)
Antibody	Mouse monoclonal α-GAPDH	abcam	ab8245; RRID:AB_2107448	(1:1000 dilution)
Antibody	Rabbit polyclonal α-HAX1	abcam	ab137613; RRID:AB_2847902	(1:500 dilution)
Antibody	Rabbit polyclonal α-MICU2	abcam	ab101465; RRID:AB_10711219	(1:1000 dilution)
Antibody	IRDye 680RD Goat anti-Rabbit IgG Secondary Antibody	Li-Cor	926–68071; RRID:AB_10956166	(1:2500 dilution)
Antibody	IRDye 800CW Goat anti-Mouse IgG Secondary Antibody	Li-Cor	926–32210; RRID:AB_621842	(1:2500 dilution)
Cell line (*Escherichia coli*)	BL21-CodonPlus (DE3) -RIL competent cells	Agilent	230245	Chemically competent cells
Cell line (*Homo sapiens*)	Human HAP1 Knockout Cell Lines - WT	Horizon Discovery	HZGHC81570;; RRID:CVCL_Y019	Human control cell line. The HAP1 cell line was bought directly from Horizon Discovery and has been control quality checked by the vendor.
Cell line (*Homo sapiens*)	Human HAP1 Knockout Cell Lines - CLPB	Horizon Discovery	HZGHC007326c001	Human knockout cell line. The HAP1 ClpB knockout cell line was bought directly from Horizon Discovery and has been control quality checked by the vendor.
Commercial assay or kit	ATPase Activity Kit (Colorimetric)	Innova Biosciencens	601–0120	
Commercial assay or kit	Luciferase Assay Reagent	Promega	E1483	
Chemical compound, drug	Creatine phosphate	Roche	10621722001	
Chemical compound, drug	ADP	MP Biomedicals	150260	
Chemical compound, drug	AMPPNP	Roche	10102547001	
Chemical compound, drug	ATP	Sigma-Aldrich	A3377	
Chemical compound, drug	ATPγS	Roche	10102342001	
Chemical compound, drug	cOmplete, Mini, EDTA-free Protease Inhibitor Cocktail	Sigma-Aldrich	4693159001	
Chemical compound, drug	Pepstatin A, microbial,≥90% (HPLC)	Sigma-Aldrich	P5318	
Chemical compound, drug	Protease Inhibitor Cocktail (mammalian)	Sigma-Aldrich	P8340	
Gene (*Homo sapiens*)	*CLPB*	NA	HGNC:30664	
Peptide, recombinant protein	α-synuclein fibrils	Luk et al., 2012		Preformed α-synuclein fibrils were a gift from Kelvin Luk.
Peptide, recombinant protein	β-casein	Sigma-Aldrich	C6905	
Peptide, recombinant protein	Creatine kinase	Roche	10127566001	
Peptide, recombinant protein	Firefly luciferase	Sigma-Aldrich	L9506	
Peptide, recombinant protein	Hdj1	Michalska et al., 2019		
Peptide, recombinant protein	Hsc70	Michalska et al., 2019		
Peptide, recombinant protein	Hsp104	DeSantis et al., 2012 and Jackrel et al., 2014		
Peptide, recombinant protein	Lysozyme	Sigma-Aldrich	L6876	
Peptide, recombinant protein	_MPP_Skd3	This study		Full description can be found in Materials and methods: Purification of Skd3
Peptide, recombinant protein	_MPP_Skd3^K387A^	This study		Full description can be found in Materials and methods: Purification of Skd3
Peptide, recombinant protein	_MPP_Skd3^E455Q^	This study		Full description can be found in Materials and methods: Purification of Skd3
Peptide, recombinant protein	_MPP_Skd3^Y430A^	This study		Full description can be found in Materials and methods: Purification of Skd3
Peptide, recombinant protein	_PARL_Skd3	This study		Full description can be found in Materials and methods: Purification of Skd3
Peptide, recombinant protein	_PARL_Skd3^K387A^	This study		Full description can be found in Materials and methods: Purification of Skd3
Peptide, recombinant protein	_PARL_Skd3^E455Q^	This study		Full description can be found in Materials and methods: Purification of Skd3
Peptide, recombinant protein	_PARL_Skd3^Y430A^	This study		Full description can be found in Materials and methods: Purification of Skd3
Peptide, recombinant protein	_PARL_Skd3^T268M^	This study		Full description can be found in Materials and methods: Purification of Skd3
Peptide, recombinant protein	_PARL_Skd3^R475Q^	This study		Full description can be found in Materials and methods: Purification of Skd3
Peptide, recombinant protein	_PARL_Skd3^A591V^	This study		Full description can be found in Materials and methods: Purification of Skd3
Peptide, recombinant protein	_PARL_Skd3^R650P^	This study		Full description can be found in Materials and methods: Purification of Skd3
Peptide, recombinant protein	Skd3_ANK_	This study		Full description can be found in Materials and methods: Purification of Skd3
Peptide, recombinant protein	Skd3_NBD_	This study		Full description can be found in Materials and methods: Purification of Skd3
Recombinant DNA reagent	Hdj1 in pE-SUMO	Michalska et al., 2019		
Recombinant DNA reagent	Hsc70 in pE-SUMO	Michalska et al., 2019		
Recombinant DNA reagent	Hsp104 in pNOTAG	DeSantis et al., 2012 and Jackrel et al., 2014		
Recombinant DNA reagent	_MPP_Skd3 in pMAL C2 with TEV site	This study		Full description can be found in Materials and methods: Purification of Skd3
Recombinant DNA reagent	_MPP_Skd3^K387A^ in pMAL C2 with TEV site	This study		Full description can be found in Materials and methods: Purification of Skd3
Recombinant DNA reagent	_MPP_Skd3^E455Q^ in pMAL C2 with TEV site	This study		Full description can be found in Materials and methods: Purification of Skd3
Recombinant DNA reagent	_MPP_Skd3^Y430A^ in pMAL C2 with TEV site	This study		Full description can be found in Materials and methods: Purification of Skd3
Recombinant DNA reagent	_PARL_Skd3 in pMAL C2 with TEV site	This study		Full description can be found in Materials and methods: Purification of Skd3
Recombinant DNA reagent	_PARL_Skd3^K387A^ in pMAL C2 with TEV site	This study		Full description can be found in Materials and methods: Purification of Skd3
Recombinant DNA reagent	_PARL_Skd3^E455Q^ in pMAL C2 with TEV site	This study		Full description can be found in Materials and methods: Purification of Skd3
Recombinant DNA reagent	_PARL_Skd3^Y430A^ in pMAL C2 with TEV site	This study		Full description can be found in Materials and methods: Purification of Skd3
Recombinant DNA reagent	_PARL_Skd3^T268M^ in pMAL C2 with TEV site	This study		Full description can be found in Materials and methods: Purification of Skd3
Recombinant DNA reagent	_PARL_Skd3^R475Q^ in pMAL C2 with TEV site	This study		Full description can be found in Materials and methods: Purification of Skd3
Recombinant DNA reagent	_PARL_Skd3^A591V^ in pMAL C2 with TEV site	This study		Full description can be found in Materials and methods: Purification of Skd3
Recombinant DNA reagent	_PARL_Skd3^R650P^ in pMAL C2 with TEV site	This study		Full description can be found in Materials and methods: Purification of Skd3
Recombinant DNA reagent	Skd3_ANK_ in pMAL C2 with TEV site	This study		Full description can be found in Materials and methods: Purification of Skd3
Recombinant DNA reagent	Skd3_NBD_ in pMAL C2 with TEV site	This study		Full description can be found in Materials and methods: Purification of Skd3
Sequence-based reagent	ClpA (*Escherichia coli*)	This study	UniProtKB:P0ABH9	Full description can be found in Materials and methods: Purification of Skd3
Sequence-based reagent	ClpB (*Escherichia coli*)	This study	UniProtKB:P63284	Full description can be found in Materials and methods: Purification of Skd3
Sequence-based reagent	ClpC (*Staphylococcus aureus*)	This study	UniProtKB:Q2G0P5	Full description can be found in Materials and methods: Purification of Skd3
Sequence-based reagent	Hsp104 (*Saccharomyces cerevisiae*)	This study	UniProtKB:P31539	Full description can be found in Materials and methods: Purification of Skd3
Sequence-based reagent	Hsp78 (*Saccharomyces cerevisiae*)	This study	UniProtKB:P33416	Full description can be found in Materials and methods: Purification of Skd3
Sequence-based reagent	HAX1 (*Homo sapiens*)	This study	UniProtKB:O00165	Full description can be found in Materials and methods: Purification of Skd3
Sequence-based reagent	Skd3 (*Ailuropoda melanoleuca*)	This study		Full description can be found in Materials and methods: Purification of Skd3
Sequence-based reagent	Skd3 (*Anolis carolinensis*)	This study		Full description can be found in Materials and methods: Purification of Skd3
Sequence-based reagent	Skd3 (*Bos taurus*)	This study		Full description can be found in Materials and methods: Purification of Skd3
Sequence-based reagent	Skd3 (*Callithrix jacchus*)	This study		Full description can be found in Materials and methods: Purification of Skd3
Sequence-based reagent	Skd3 (*Callorhinus ursinus*)	This study		Full description can be found in Materials and methods: Purification of Skd3
Sequence-based reagent	Skd3 (*Canis lupus*)	This study		Full description can be found in Materials and methods: Purification of Skd3
Sequence-based reagent	Skd3 (*Capra hircus*)	This study		Full description can be found in Materials and methods: Purification of Skd3
Sequence-based reagent	Skd3 (*Carlito syrichta*)	This study		Full description can be found in Materials and methods: Purification of Skd3
Sequence-based reagent	Skd3 (*Cebus capucinus*)	This study		Full description can be found in Materials and methods: Purification of Skd3
Sequence-based reagent	Skd3 (*Ceratotherium simum*)	This study		Full description can be found in Materials and methods: Purification of Skd3
Sequence-based reagent	Skd3 (*Cercocebus atys*)	This study		Full description can be found in Materials and methods: Purification of Skd3
Sequence-based reagent	Skd3 (*Chlorocebus sabaeus*)	This study		Full description can be found in Materials and methods: Purification of Skd3
Sequence-based reagent	Skd3 (*Colobus angolensis*)	This study		Full description can be found in Materials and methods: Purification of Skd3
Sequence-based reagent	Skd3 (*Danio rerio*)	This study		Full description can be found in Materials and methods: Purification of Skd3
Sequence-based reagent	Skd3 (*Equus asinus*)	This study		Full description can be found in Materials and methods: Purification of Skd3
Sequence-based reagent	Skd3 (*Equus caballus*)	This study		Full description can be found in Materials and methods: Purification of Skd3
Sequence-based reagent	Skd3 (*Equus przewalskii*)	This study		Full description can be found in Materials and methods: Purification of Skd3
Sequence-based reagent	Skd3 (*Felis catus*)	This study		Full description can be found in Materials and methods: Purification of Skd3
Sequence-based reagent	Skd3 (*Geospiza fortis*)	This study		Full description can be found in Materials and methods: Purification of Skd3
Sequence-based reagent	Skd3 (*Gorilla gorilla*)	This study		Full description can be found in Materials and methods: Purification of Skd3
Sequence-based reagent	Skd3 (*Gulo gulo*)	This study		Full description can be found in Materials and methods: Purification of Skd3
Sequence-based reagent	Skd3 (*Grammomys surdaster*)	This study		Full description can be found in Materials and methods: Purification of Skd3
Sequence-based reagent	Skd3 (*Homo sapiens)*	This study	UniProtKB:Q9H078	Full description can be found in Materials and methods: Purification of Skd3
Sequence-based reagent	Skd3 (*Macaca fascicularis*)	This study		Full description can be found in Materials and methods: Purification of Skd3
Sequence-based reagent	Skd3 (*Macaca mulatta*)	This study		Full description can be found in Materials and methods: Purification of Skd3
Sequence-based reagent	Skd3 (*Macaca nemestrina*)	This study		Full description can be found in Materials and methods: Purification of Skd3
Sequence-based reagent	Skd3 (*Mandrillus leucophaeus*)	This study		Full description can be found in Materials and methods: Purification of Skd3
Sequence-based reagent	Skd3 (*Microcebus murinus*)	This study		Full description can be found in Materials and methods: Purification of Skd3
Sequence-based reagent	Skd3 (*Microtus ochrogaster*)	This study		Full description can be found in Materials and methods: Purification of Skd3
Sequence-based reagent	Skd3 (*Monosigia brevicollis*)	This study		Full description can be found in Materials and methods: Purification of Skd3
Sequence-based reagent	Skd3 (*Mus musculus*)	This study		Full description can be found in Materials and methods: Purification of Skd3
Sequence-based reagent	Skd3 (*Mustela putorius*)	This study		Full description can be found in Materials and methods: Purification of Skd3
Sequence-based reagent	Skd3 (*Nomascus leucogenys*)	This study		Full description can be found in Materials and methods: Purification of Skd3
Sequence-based reagent	Skd3 (*Nothobranchius rachovii*)	This study		Full description can be found in Materials and methods: Purification of Skd3
Sequence-based reagent	Skd3 (*Odobenus rosmarus*)	This study		Full description can be found in Materials and methods: Purification of Skd3
Sequence-based reagent	Skd3 (*Orycteropus afer*)	This study		Full description can be found in Materials and methods: Purification of Skd3
Sequence-based reagent	Skd3 (*Pan paniscus*)	This study		Full description can be found in Materials and methods: Purification of Skd3
Sequence-based reagent	Skd3 (*Pan troglodyte*)	This study		Full description can be found in Materials and methods: Purification of Skd3
Sequence-based reagent	Skd3 (*Panthera tigris*)	This study		Full description can be found in Materials and methods: Purification of Skd3
Sequence-based reagent	Skd3 (*Papio anubis*)	This study		Full description can be found in Materials and methods: Purification of Skd3
Sequence-based reagent	Skd3 (*Piliocolobus tephrosceles*)	This study		Full description can be found in Materials and methods: Purification of Skd3
Sequence-based reagent	Skd3 (*Pongo abelii*)	This study		Full description can be found in Materials and methods: Purification of Skd3
Sequence-based reagent	Skd3 (*Propithecus coquereli*)	This study		Full description can be found in Materials and methods: Purification of Skd3
Sequence-based reagent	Skd3 (*Puma concolor*)	This study		Full description can be found in Materials and methods: Purification of Skd3
Sequence-based reagent	Skd3 (*Rattus norvegicus*)	This study		Full description can be found in Materials and methods: Purification of Skd3
Sequence-based reagent	Skd3 (*Rhinopithecus bieti*)	This study		Full description can be found in Materials and methods: Purification of Skd3
Sequence-based reagent	Skd3 (*Rhinopithecus roxellana*)	This study		Full description can be found in Materials and methods: Purification of Skd3
Sequence-based reagent	Skd3 (*Rousettus aegyptiacus*)	This study		Full description can be found in Materials and methods: Purification of Skd3
Sequence-based reagent	Skd3 (*Sus scrofa*)	This study		Full description can be found in Materials and methods: Purification of Skd3
Sequence-based reagent	Skd3 (*Theropithecus gelada*)	This study		Full description can be found in Materials and methods: Purification of Skd3
Sequence-based reagent	Skd3 (*Trachymyrmex septentrionalis*)	This study		Full description can be found in Materials and methods: Purification of Skd3
Sequence-based reagent	Skd3 (*Trichinella papuae*)	This study		Full description can be found in Materials and methods: Purification of Skd3
Sequence-based reagent	Skd3 (*Ursus arctos*)	This study		Full description can be found in Materials and methods: Purification of Skd3
Sequence-based reagent	Skd3 (*Ursus maritimus*)	This study		Full description can be found in Materials and methods: Purification of Skd3
Sequence-based reagent	Skd3 (*Xenopus laevis*)	This study		Full description can be found in Materials and methods: Purification of Skd3
Sequence-based reagent	Skd3 (*Zalophus californianus*)	This study		Full description can be found in Materials and methods: Purification of Skd3
Software, algorithm	BOXSHADE	Hofmann and Baron, 1996		
Software, algorithm	Clusal Omega	Madeira et al., 2019		
Software, algorithm	EXPASY ProtParam	Kyte and Doolittle, 1982; Wilkins et al., 1999		
Software, algorithm	FigTree	Rambaut, 2012		
Software, algorithm	Gene Ontology (GO)	Ashburner et al., 2000; Mi et al., 2019; The Gene Ontology Consortium, 2019		
Software, algorithm	IUPRED	Mészáros et al., 2018; Mészáros et al., 2009		
Software, algorithm	PhyloPic	http://phylopic.org/		
Software, algorithm	Prism 8	GraphPad		
Software, algorithm	WebLogo	https://weblogo. berkeley.edu/logo.cgi		
