# Supplementary material for: Skd3 (human ClpB) is a potent mitochondrial protein disaggregase that is inactivated by 3-methylglutaconic aciduria-linked mutations
Source: eLife. 2020 Jun 23;9:e55279. doi: 10.7554/eLife.55279 (PMC7343390; doi:10.7554/eLife.55279)
Supplement: Supplementary file 1. — Alignment of Skd3 protein from diverse metazoan lineages. Alignment was constructed using Clustal Omega. Alignment shows high level of conservation of Skd3 among species. H. sapiens, G. gorilla, and C. jacchus Skd3 have an additional insertion in the ankyrin-repeat domain that is not conserved in the other species. This alignment was used to generate the phylogenetic tree in Figure 1B. The protozoan M. brevicollis Skd3 sequence was included in the alignment for reference. MTS (mitochondrial-targeting sequence, ANK (ankyrin-repeat domain), NBD (nucleotide-binding domain), and CTD (C-terminal domain). [file elife-55279-supp1.docx]

**
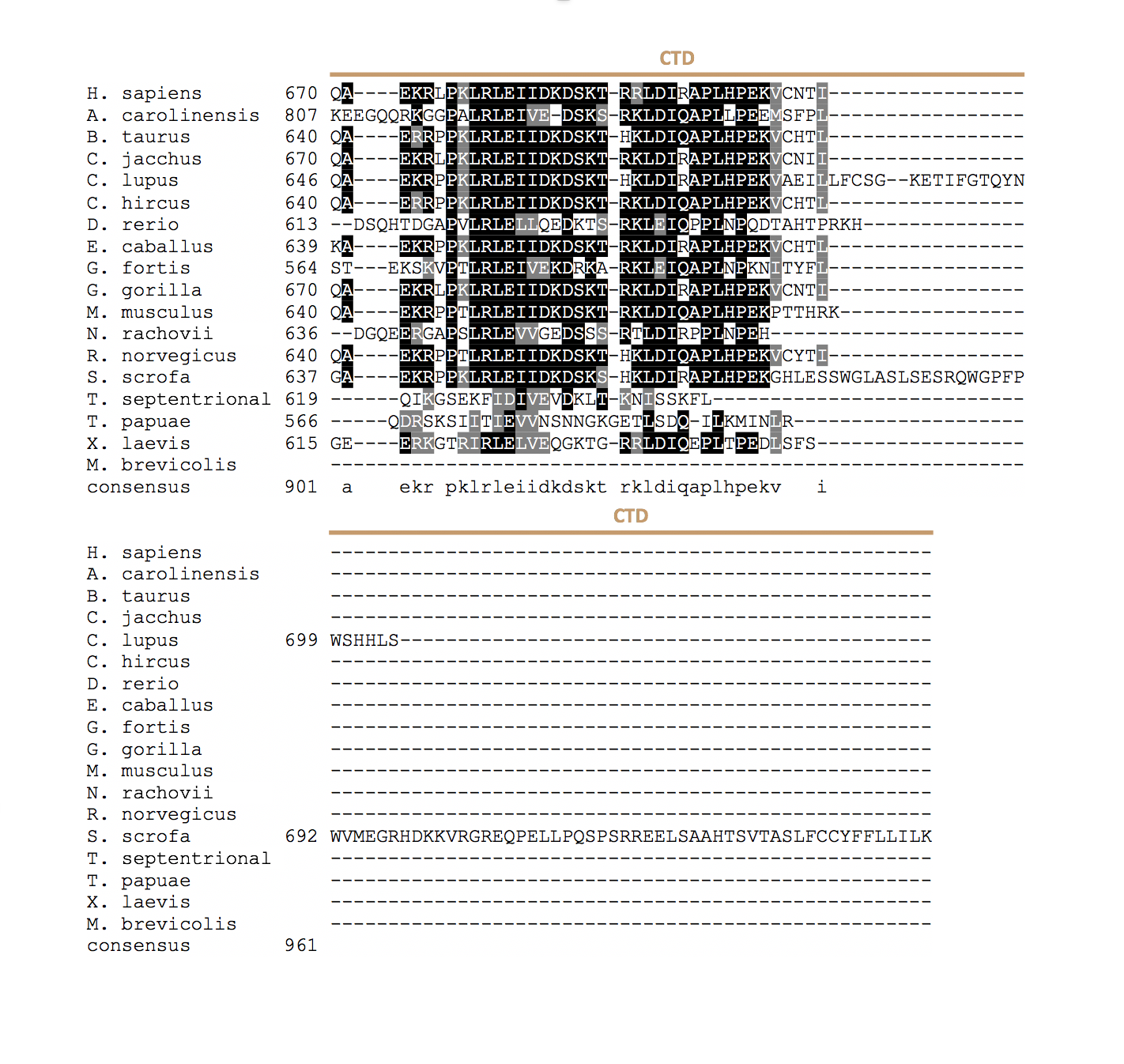
**

**Supplementary File 1. Alignment of Skd3 to diverse metazoan lineages shows conservation of key motifs and domains.** Alignment of Skd3 protein from diverse metazoan lineages. Alignment was constructed using Clustal Omega. Alignment shows high level of conservation of Skd3 among species. *H. sapiens*, *G. gorilla*, and *C. jacchus* Skd3 have an additional insertion in the ankyrin-repeat domain that is not conserved in the other species. This alignment was used to generate the phylogenetic tree in Figure 1B. The protozoan *M. brevicollis* Skd3 sequence was included in the alignment for reference. MTS (mitochondrial-targeting sequence, ANK (ankyrin-repeat domain), NBD (nucleotide-binding domain), and CTD (C-terminal domain).
